# Decision-making in stimulant and opiate addicts in protracted abstinence: evidence from computational modeling with pure users

**DOI:** 10.3389/fpsyg.2014.00849

**Published:** 2014-08-12

**Authors:** Woo-Young Ahn, Georgi Vasilev, Sung-Ha Lee, Jerome R. Busemeyer, John K. Kruschke, Antoine Bechara, Jasmin Vassileva

**Affiliations:** ^1^Virginia Tech Carilion Research Institute, Virginia TechRoanoke, VA, USA; ^2^Bulgarian Addictions InstituteSofia, Bulgaria; ^3^Department of Psychological and Brain Sciences, Indiana UniversityBloomington, IN, USA; ^4^Department of Psychology, University of Southern CaliforniaLos Angeles, CA, USA; ^5^Brain and Creativity Institute, University of Southern CaliforniaLos Angeles, CA, USA; ^6^Department of Psychiatry, Virginia Commonwealth University School of MedicineRichmond, VA, USA

**Keywords:** addiction, decision-making, computational modeling, heroin, amphetamine, protracted abstinence, Bayesian data analysis, Widely Applicable Information Criterion (WAIC)

## Abstract

Substance dependent individuals (SDI) often exhibit decision-making deficits; however, it remains unclear whether the nature of the underlying decision-making processes is the same in users of different classes of drugs and whether these deficits persist after discontinuation of drug use. We used computational modeling to address these questions in a unique sample of relatively “pure” amphetamine-dependent (*N* = 38) and heroin-dependent individuals (*N* = 43) who were currently in protracted abstinence, and in 48 healthy controls (HC). A Bayesian model comparison technique, a simulation method, and parameter recovery tests were used to compare three cognitive models: (1) Prospect Valence Learning with decay reinforcement learning rule (PVL-DecayRI), (2) PVL with delta learning rule (PVL-Delta), and (3) Value-Plus-Perseverance (VPP) model based on Win-Stay-Lose-Switch (WSLS) strategy. The model comparison results indicated that the VPP model, a hybrid model of reinforcement learning (RL) and a heuristic strategy of perseverance had the best *post-hoc* model fit, but the two PVL models showed better simulation and parameter recovery performance. Computational modeling results suggested that overall all three groups relied more on RL than on a WSLS strategy. Heroin users displayed reduced loss aversion relative to HC across all three models, which suggests that their decision-making deficits are longstanding (or pre-existing) and may be driven by reduced sensitivity to loss. In contrast, amphetamine users showed comparable cognitive functions to HC with the VPP model, whereas the second best-fitting model with relatively good simulation performance (PVL-DecayRI) revealed increased reward sensitivity relative to HC. These results suggest that some decision-making deficits persist in protracted abstinence and may be mediated by different mechanisms in opiate and stimulant users.

## Introduction

Drug addiction is a chronic relapsing brain disease, characterized by compulsive drug seeking and use despite negative consequences in major life domains (Goldstein and Volkow, 2011). Substance dependent individuals (SDI) are commonly characterized by decision-making deficits, both on laboratory tasks and in real life, manifested by lack of judgment and reduced concern for the consequences of their actions. What remains unknown, however, is whether these decision-making deficits are equally represented across addictions to different classes of drugs.

Current theories consider addiction to different classes of drugs as a unitary phenomenon, in part based on evidence that most drugs of abuse act on the mesocortico/mesolimbic dopamine (DA) system (Wise, 1978; Di Chiara and Imperato, 1988; Robinson and Berridge, 1993). More recently, however, animal and human studies have begun to reveal important cognitive and neurobiological differences between addictions to different classes of drugs, such as stimulants and opiates (Pettit et al., 1984; Rogers et al., 1999; Ersche et al., 2005b; Badiani et al., 2011). It is now well known that these two classes of drugs act on different mechanisms of DA modulation (Kreek et al., 2002, 2012). DA transmission mediates self-administration of stimulants, but not of opiates; in contrast, the μ-opiate receptor plays an important role for opiate, but not for stimulant self-administration (Badiani et al., 2011). Further, genetic studies reveal minimal overlap of genes associated with stimulant and opiate addiction (Yuferov et al., 2010).

Preclinical studies reveal notable differences between stimulants and opiates, which exert fundamentally different behavioral effects, such that stimulants produce arousing and activating effects, whereas opiates produce mixed inhibitory and excitatory effects (Stewart et al., 1984). Of note, the rewarding effects of stimulant self-administrations are greater in new and arousing environments than in familiar and safe environments, whereas the opposite is observed with the sedative effects of opiates (Caprioli et al., 2008). Further, the neural pathway activated by aversive stimuli from lateral habenula to rostromedial tegmental nucleus (RMTg) is affected by opiates, but not by stimulants (Lecca et al., 2011).

In contrast, studies comparing neurocognitive performance of human stimulant and opiate users have shown mixed results. Some studies reveal distinct performance patterns in stimulant vs. opiate users. Rogers et al. (1999) report that amphetamine users perform worse than healthy individuals on the Cambridge Gambling Task, whereas opiate users display intact performance on this decision task. In addition, duration of drug abuse was associated with suboptimal decision-making in stimulant users, but not in opiate users. In another study (Ornstein et al., 2000), amphetamine and heroin abusers were characterized by different attentional shifting deficits, with amphetamine users being impaired on the extra-dimensional (ED) and heroin users on the intra-dimensional (ID) shift component of the task. Also, cocaine users, but not heroin users show deficits in response inhibition (Verdejo-Garcia et al., 2007b). In contrast, other studies reveal comparable neurocognitive profiles between users of these two classes of drugs. Both cocaine and heroin users show higher discounting of delayed rewards compared to alcohol users and healthy individuals (Kirby and Petry, 2004). Further, on a task measuring reflection impulsivity, both amphetamine- and opiate-dependent individuals sample less information and perform worse than healthy individuals (Clark et al., 2006).

Decision-making is one of the neurocognitive domains on which SDI are commonly impaired. It is typically indexed in the laboratory with tasks that simulate real-life decision-making such as the Iowa Gambling Task (IGT) (Bechara et al., 1994), on which SDI often select choices that yield high immediate gains but have higher future losses (Grant et al., 2000; Bechara et al., 2001; Bolla et al., 2003; Bechara and Martin, 2004; Gonzalez et al., 2007; Vassileva et al., 2007a; Verdejo-Garcia et al., 2007a). Decision-making deficits among SDI are of immediate practical concern, in light of their associations with HIV risk behaviors (Gonzalez et al., 2005) and clinical outcomes such as abstinence (Passetti et al., 2008). The IGT is a complex task and poor behavioral performance could be the result of deficits in various distinct neurocognitive processes, such as hypersensitivity to reward and/or hyposensitivity to losses, failure to learn from past outcomes and losses, and/or erratic and impulsive response style. In a series of studies, Busemeyer et al. (Busemeyer and Stout, 2002; Stout et al., 2004; Yechiam et al., 2005; Ahn et al., 2008) have developed mathematical models of the task that capture the complex interplay of cognitive and motivational processes involved in decision-making. The use of such models allows one to decompose behavioral performance on the task into distinct cognitive, motivational, and response processes, thereby providing a fine-grained analysis of the underlying decision-making processes and characterizing more precisely the decision-making deficits of different clinical groups. This approach yields quantifiable parameter estimates of such processes, which have been successfully mapped in various clinical populations including cocaine users, cannabis users, alcohol users, individuals with Asperger's disease, Huntington's disease, schizophrenia, and bipolar disorder (for a review, see Yechiam et al., 2005), as well as in eating disorders (Chan et al., 2014) and patients with HIV (Vassileva et al., 2013). Studies applying this approach show that although behavioral performance may be similar across different clinical groups, the cognitive processes that underlie these behavioral profiles may vary across groups in clinically meaningful ways.

The widespread polysubstance-dependence among SDI significantly complicates attempts to dissociate pre-existing biological or personality characteristics from the effects of chronic use of different classes of drugs on neurocognitive functioning (Fernández-Serrano et al., 2011; Gorodetzky et al., 2011; Baldacchino et al., 2012). Further, we still know very little about the reversibility of the observed neurocognitive deficits with abstinence, given that with few exceptions (Ersche et al., 2005a,b; Clark et al., 2006) most studies to date have focused on current drug users or on SDI who have been abstinent for rather brief periods of time. The chronic relapsing nature of addiction suggests that some of the neurocognitive deficits, particularly those in decision-making, may persist with abstinence and may be critically implicated in increased susceptibility to relapse. In order to better understand the brain's recovery of function with protracted abstinence and to refine treatment interventions at different stages of the addiction cycle, it is crucial to get a better understanding of the specificity and the persistence of the neurocognitive deficits observed in drug users.

To address these challenges, we conducted the current study in Bulgaria, where polysubstance dependence is still relatively uncommon and where we have access to a unique population of fairly “pure” (monosubstance-dependent) amphetamine and heroin users who meet lifetime DSM-IV criteria for amphetamine or heroin dependence. The heroin epidemic in Bulgaria started in the early 1990s after the end of communism, when Bulgaria became a key transit country for heroin trafficking due to its strategic geographical position on the “Balkan Drug Route,” one of the main routes for international drug traffic from South-West Asia to Western Europe. Estimates show that at times up to 80% of heroin used in Western Europe passes through this route (European Monitoring Center for Drugs and Drug Addiction, 2011). The heroin epidemic reached its peak in 1997–1998, after which it plateaued. In the early 2000s, there were an estimated 20–30,000 regular heroin addicts in Bulgaria (population of ~7,476,000 people), which number has remained steady over the last decade, with a recent trend for a slight decline. Typically, heroin addicts belong to a cohort of somewhat aging addicts, ~30 years of age. In contrast, the amphetamine epidemic in Bulgaria started more recently in the new millennium when Bulgaria became a major center for production of synthetic amphetamine-type stimulants and is currently one of the top five highest-prevalence countries in Europe (European Monitoring Center for Drugs and Drug Addiction, 2011). Hence, amphetamine users are typically younger—normally in their late teens or early 20s. Notably, few SDI use the two types of drugs concurrently.

We compared the decision-making performance of heroin and amphetamine users to that of healthy controls (HC) without any history of substance dependence. We followed these behavioral analyses by applying a computational modeling approach, in order to better characterize their decision-making styles and to disentangle the distinct neurocognitive processes underlying the decision-making performance of heroin and amphetamine users. The modeling results and their interpretations depend on which model we use. Therefore, we first identified the best-fitting model by comparing three existing computational models using a Bayesian model comparison technique, a simulation method, and parameter recovery tests (see Materials and Methods below for more details). Then, we compared groups in a Bayesian way using the best-fitting model, but also tested whether we would observe similar group differences with the other models. Based on previous animal and human studies, we hypothesized that amphetamine and heroin users would show distinct decision-making profiles. Specifically, we expected that amphetamine users would show increased reward sensitivity and heroin users would show reduced loss aversion compared to HC (Spotts and Shontz, 1980; Stewart et al., 1984; Kreek et al., 2002).

In light of the growing evidence for the relationship of externalizing and internalizing personality traits and disorders with decision-making and drug addiction, in exploratory analyses we considered the relationship between impulsivity and psychopathy (externalizing spectrum) and depression and anxiety (internalizing spectrum) with decision-making. We hypothesized that externalizing but not internalizing traits and states would be associated with compromised decision-making.

## Materials and methods

### Participants

Study participants included 129 individuals, enrolled in a larger study of impulsivity in heroin and amphetamine users in Sofia, Bulgaria. Potential participants were recruited via flyers placed at substance abuse clinics, cafes, bars, and night clubs in Sofia and screened via telephone and in-person on their medical and substance use histories. SDI had lifetime DSM-IV histories of opiate or stimulant dependence. The current study included primarily monosubstance-dependent users with no history of dependence on alcohol or any drug other than opiates or stimulants (with the exception of nicotine, caffeine, and/or past cannabis dependence). Demographically similar individuals with no history of substance dependence were included as controls. Study participants included 38 amphetamine users, 43 heroin users, and 48 HC. Most of the heroin and amphetamine users were in protracted abstinence at the time of testing (~2.9 years on average since they last met DSM-IV criteria for substance dependence, minimum 3 months post discontinuation of drug use). Among the 38 amphetamine users, 11 were in early (<12 months of abstinence) full (*n* = 9; 24%) or partial (*n* = 2; 5%) remission and 27 were in sustained (>12 months of abstinence) full (*n* = 25; 66%) or partial (*n* = 2; 5%) remission. Among the 43 heroin users, 12 (28%) were in early full remission, 30 (70%) were in sustained full and one (2%) was in sustained partial remission.

Inclusion criteria consisted of age between 18 and 50 years, minimum of 8 years of formal education, ability to speak and read Bulgarian, estimated IQ greater than 80, negative breathalyzer test for alcohol and negative rapid urine toxicology screen for opiates, cannabis, amphetamines, methamphetamines, benzodiazepines, barbiturates, cocaine, MDMA, and methadone. Exclusion criteria included history of neurologic illness or injury, history of psychotic disorders, and current opioid substitution therapy (OST). All participants were HIV-seronegative, as verified by rapid HIV test. All participants provided written informed consent. Study procedures were approved by the Institutional Review Boards of the University of Illinois at Chicago and the Medical University in Sofia on behalf of the Bulgarian Addictions Institute.

### Assessment

History of substance abuse and dependence was determined using the Structured Clinical Interview for DSM-IV Substance Abuse Module (SCID-SAM; First et al., 1996). The Raven's Progressive Matrices was administered to index estimated IQ. For the exploratory analyses, the Barratt Impulsiveness Scale—11th revision (BIS-11; Patton and Stanford, 1995) indexed the personality trait of impulsivity. Psychopathy was assessed with the Psychopathy Checklist: Screening Version (PCL:SV; Hart et al., 1995). Current depression was assessed with the [Beck Depression Inventory-II (BDI-II); Beck et al., 1996] and anxiety with the [State-Trait Anxiety Inventory (STAI); Spielberger and Gorsuch, 1983]. For the exploratory analyses, we also tabulated several substance use characteristics including number of years of drug use, length of abstinence from the primary drug of dependence, number of DSM-IV criteria met for the primary drug of dependence, severity of nicotine dependence, and history of past cannabis dependence.

### Iowa gambling task

Decision-making was measured with the computerized IGT (Bechara et al., 1994, 2001), arguably the most popular decision task in the addiction literature. The task requires participants to select cards from one of four decks with the goal of maximizing profits. Unbeknownst to participants, two of the decks (decks C and D) are *advantageous (“good”)* and two (decks A and B) are *disadvantageous (“bad”)* in terms of their long-term payoffs. The frequencies of punishment also vary across decks such that punishment is more frequent in decks A and C (50%) than in decks B and D (10%). In the modified version of the IGT (Bechara et al., 2001) used in the current study, each deck has up to 60 cards and the amounts of net gains or losses increased incrementally in every block of 10 cards. For example, the net loss of decks A and B in the first block of 10 cards is -$250, but across every block it goes up with $150 until it reaches $1000 in the sixth block. Similarly, the net gain of decks C and D goes up from $250 in the first block to $375 in the sixth block, with an increment of $25 in each block of 10 cards. The frequencies of punishment are identical to those in the original IGT version. Participants have to learn the task contingencies by trial-and-error. Healthy participants typically learn to select cards from the advantageous decks as the task progresses, thereby achieving a higher cumulative reward value. Behavioral performance analyses were based on the total net score, calculated by subtracting the number of disadvantageous deck selections from the number of advantageous deck selections. Trial-by-trial choice data of the HC, amphetamine, and heroin groups are available at http://figshare.com/articles/IGT_raw_data_Ahn_et_al_2014_Frontiers_in_Psychology/1101324.

### Computational modeling of decision-making

From a statistical perspective, the IGT is a four-armed bandit problem (Berry and Fristedt, 1985), a special case of reinforcement learning (RL) problems in which an agent needs to learn an environment by choosing actions and experiencing the outcomes of those actions. Poor performance on the IGT can be due to a number of distinct underlying neurocognitive processes such as poor learning/memory, hypersensitivity to reward, hyposensitivity to loss, or response inconsistency. In order to better characterize behavioral performance on the IGT and to disentangle the distinct neurocognitive processes underlying the performance of pure heroin and amphetamine users on the task, we next used the *computational modeling approach* (Busemeyer and Stout, 2002; Yechiam et al., 2005; Ahn et al., 2008).

We compared three of the most promising models of the IGT according to the literature (e.g., Ahn et al., 2008, 2011; Steingroever et al., 2013, 2014; Worthy et al., 2013b): the Prospect Valence Learning (PVL) model with delta learning rule (PVL-Delta) (Ahn et al., 2008), the PVL model with decay reinforcement learning rule (PVL-DecayRI) (Ahn et al., 2008, 2011), and the Value-Plus-Perseverance model (VPP) (Worthy et al., 2013b). We used Watanabe-Akaike Information Criterion (also called Widely Applicable Information Criterion; WAIC) (Watanabe, 2010) to compare the *post-hoc* fits of models. We also used a simulation method to examine whether a model with estimated parameters can generate the observed choice pattern (Ahn et al., 2008; Steingroever et al., 2014). We describe the mathematical details of all models, which are also available in the previous publication (Worthy et al., 2013b) as well as WAIC and the simulation method below.

#### Prospect valence learning (PVL) models (PVL-Delta and PVL-DecayRI)

The PVL models have three components. The PVL-Delta and PVL-DecayRI models are identical except that they use different learning rules. First, the outcome evaluation follows the Prospect utility function that has diminishing sensitivity to increases in magnitude and different outcome sensitivity to losses vs. gains (i.e., loss aversion). The utility, *u*(*t*) on trial *t* of each net outcome *x*(*t*) is expressed as:

(1) $$u(t)=\begin{array}{l} \begin{array}{l} x{\left(t\right)}^{\alpha }\;if\;x(t)\geq 0\\ -\lambda |x(t){|}^{\alpha }\;if\;x(t)<0 \end{array} \end{array}$$

Here α (shape parameter, 0 < α < 2) governs the shape of the utility function and λ (loss aversion parameter, 0 < λ < 10) determines the sensitivity to losses compared to gains. Net outcomes were scaled (all payoff outcomes were divided by a fixed number) for cognitive modeling so that the median highest net gain across subjects in the first block of 10 trials becomes 1 and the largest net loss becomes −11.5 (Busemeyer and Stout, 2002). If an individual has a high value of α, it indicates that he/she has greater sensitivity to feedback outcomes than an individual with a low value of α. Here, we extended the upper bound of α to be greater than 1 as some individuals may have very high values of α (e.g., Fridberg et al., 2010). A value of λ less than 1 indicates that the individual is more sensitive to gains than to losses while a value of λ greater than 1 indicates that he/she is more sensitive to losses than to gains.

Based on the outcome of the chosen option, the expectancies of the decks were computed using a learning rule. Previous studies consistently show that the decay-reinforcement learning (decayRI; Erev and Roth, 1998) has better *post-hoc* model-fits than the delta (Rescorla-Wagner; Rescorla and Wagner, 1972) rule on the IGT (Yechiam and Busemeyer, 2005, 2008; Ahn et al., 2008) but the delta rule outperforms the decayRI learning rule in simulation tests (Ahn et al., 2008; Steingroever et al., 2014). In the decayRI learning rule, the expectancies of all decks are discounted on each trial and then the expectancy of the chosen deck is updated by the current outcome utility:

(2) $$E_{j}(t+1)=A\cdot E_{j}(t)+\delta_{j}(t)\cdot u(t)$$

*A* (*recency parameter/learning rate*, 0 < *A* < 1) determines how much the past expectancy is discounted. δ_*j*_(*t*) is a dummy variable which is 1 if deck *j* is chosen and 0 otherwise. On the other hand, in the delta rule, the expectancy of only the selected deck is updated and the expectancies of the other decks remain unchanged:

(3) $$E_{j}(t+1)=E_{j}(t)+A\cdot \delta_{j}(t)\cdot (u(t)-E_{j}(t))$$

*A* determines how much weight is placed on past experiences of the chosen deck vs. the most recent selection from the deck. A low learning rate indicates that the most recent outcome has a small influence on the expectancy and forgetting is more gradual. A high learning rate indicates that the recent outcome has a large influence on the expectancy of the chosen deck and forgetting is more rapid. Note that we used the same symbol (*A*) for the learning models in the two PVL models, but A has different meaning in each learning model (i.e., recency for the DecayRI and learning rate for the Delta model).

The softmax choice rule (Luce, 1959) was then used to compute the probability of choosing each deck *j*. θ (sensitivity) governs the degree of exploitation vs. exploration:

(4) $$\mathrm{Pr}[D(t+1)=j]=\frac{e^{\theta \cdot E_{j}(t+1)}}{\sum_{k=1}^{4}e^{\theta \cdot E_{k}(t+1)}}$$

θ is assumed to be trial-independent and was set to 3^*c*^ − 1 (Yechiam and Ert, 2007; Ahn et al., 2008). *c* is a *consistency parameter* (choice sensitivity), which was limited from 0 to 5 so that the sensitivity ranges from 0 (random) to 242 (almost deterministic).

#### Value-plus-perseverance model

Recent work suggests that participants often use a simple win-stay-lose-switch (WSLS) or perseverative strategy on the IGT, which cares only about the very last trial's information for making a decision on the current trial (Worthy et al., 2013a). Worthy et al. (2013a) compared the PVL-DecayRI and the WSLS models of the IGT using model-comparison methods. They showed that the PVL-DecayRI had the best model fits for about half of the subjects, whereas the WSLS model was the best-fitting model for the other half. Based on these findings, Worthy et al. (2013b) developed a VPP model, which is a hybrid model (e.g., Daw et al., 2011) of the PVL-Delta and a heuristic strategy of perseverance. Worthy et al. (2013b) showed that the VPP model showed the best *post-hoc* model-fits and simulation performance compared to other models for the IGT in healthy individuals.

The VPP model assumes that a participant keeps track of deck expectancies *E*_*j*_(*t*) and perseverance strengths (*P*_*j*_(*t*)). The expectancies are computed by the learning rule of the PVL-Delta model (Equation 3). For the perseverance strengths of unchosen decks on the current trial *t*, *P*_*j*_ (*t* + 1) = *k* · *P*_*j*_ (*t*). For the chosen deck:

(5) $$P_{j}(t+1)=\begin{array}{l} \begin{array}{l} k\cdot P_{j}(t)+\varepsilon_{p}\;if\;x(t)\geq 0\\ k\cdot P_{j}(t)+\varepsilon_{n}\;if\;x(t)<0 \end{array} \end{array}.$$

Here, three additional free parameters related to perseverance are introduced: *k* (0 < *k* < 1) is a decay parameter similar to A in the PVL-DecayRI model, which determines how much the perseverance strengths of all decks (including unselected decks) are decayed on each trial. ε_*p*_ and ε_*n*_ indicate the impact of gain and loss on perseverance behavior, respectively. A positive value would indicate that the feedback reinforces a tendency to persevere on the same deck on the next trial whereas a negative value would indicate that the feedback reinforces a tendency to switch from the chosen deck.

The overall value, *V*_*j*_(*t* + 1), is the weighted sum of *E*_*j*_(*t* + 1) and *P*_*j*_(*t* + 1):

(6) $$V_{j}(t+1)=\omega \cdot E_{j}(t+1)+(1-\omega )\cdot P_{j}(t+1)$$

Here ω is the RL weight (0 < ω < 1). A low value of ω would indicate that the subject would rely less on RL but more on the perseverance heuristic. A high value of ω would indicate that the subject would rely more on RL and less on the perseverance heuristic. In the VPP model, the choice probability was again using the softmax rule but with *V*_*j*_(*t* + 1):

(7) $$\mathrm{Pr}[D(t+1)=j]=\frac{e^{\theta \cdot V_{j}(t+1)}}{\sum_{k=1}^{4}e^{\theta \cdot V_{k}(t+1)}}.$$

### Statistical analyses

All data analyses were conducted using Bayesian data analysis, which has several advantages over null hypothesis significance testing (NHST) (Wagenmakers, 2007; Kruschke, 2010, 2011b, 2013): In Bayesian analysis, decisions are based on posterior probabilities of parameters (which could be model indices), not on frequentist *p* values. Unlike posterior distributions, frequentist *p* values depend on the sampling and testing intentions of the analyst. Bayesian methods also seamlessly provide posterior distributions for the type of complex hierarchical models we use here, more flexibly than deriving *p* values. For clarity and to accommodate readers more familiar with NHST, we report in parallel NHST results whenever appropriate and when there are compatible NHST approaches available. We used the posterior means of individual parameters for NHST and regression analyses. For Bayesian multiple regression and correlation analyses, we used robust regression methods so that outliers don't critically affect the inferred regression coefficients and hierarchical models, which reduces the risk of “false alarms.”

Posterior distributions on parameters are summarized by their central tendency (i.e., mean or mode) and by their highest density interval (HDI), which is the range of parameter values that span 95% of the distribution and have higher probability inside the interval than outside. The HDI can also be used to make decisions in conjunction with a region of practical equivalence (ROPE) around parameter values of interest such as zero (Kruschke, 2011a,b). If the ROPE excludes the HDI, then the ROPE'd value is said to be not credible. If the ROPE includes the HDI, then the ROPE'd value is said to be accepted for practical purposes. We leave the ROPE tacit in our analyses, as its exact size is not critical for our main conclusions. However, when the HDI excludes the value of interest (such as zero) but has a end not far from the value of interest, then a moderately large ROPE would overlap with the HDI and render the result indecisive.

#### Hierarchical Bayesian parameter estimation

The free parameters of each model were estimated using hierarchical Bayesian analysis (HBA), an emerging method in cognitive science (Lee, 2011). HBA allows for individual differences, while pooling information across individuals in a coherent way. Unlike the conventional way of parameter estimation (maximum likelihood estimation; MLE), Bayesian methods estimate full posterior distributions of parameter values rather than only point estimates. In addition, commonalities across individuals are captured by letting group tendencies inform each individual's parameter values. A recent simulation study also revealed that HBA yields much more accurate parameter estimates of the PVL-DecayRI model than non-hierarchical MLE methods. Specifically, a simulation study by Ahn et al. (2011) showed that non-hierarchical MLE estimates were often at the parameters' boundary limits (e.g., learning rate = 1) whereas parameter estimates with HBA showed much less discrepancy with actual parameter values. These results suggest that HBA would be a better method to capture individual differences in model parameters.

To perform HBA, we used a recently developed package called Stan 2.1.0 (Stan Development Team, 2014), which uses Markov chain Monte Carlo (MCMC) sampling algorithms called Hamiltonian Monte Carlo (HMC). The HMC allows efficient sampling even for complex models with multilevel structures and those with highly correlated parameters. Individual parameters were assumed to be drawn from group-level normal distributions. Normal and uniform distributions were used for the priors of normal means (μ_(.)_) and standard deviations (σ_(.)_), respectively (Wetzels et al., 2010; Steingroever et al., 2013). For parameters (say ζ for a general parameter for illustration purposes) that are bounded between 0 and 1 (e.g., *A*, *k*, ω):

(8) $$\begin{array}{l} \mu_{\xi^{\prime }}\sim \text{Normal}(0,1),\sigma_{\xi^{\prime }}\sim \text{Uniform}(0,1.5),\\ \xi^{\prime }\sim \text{Normal}(\mu_{\xi^{\prime }},\sigma_{\xi^{\prime }}),\xi =\text{Probit}(\xi^{\prime }) \end{array}$$

While Worthy et al. (2013b) set the boundary limits of ε_*p*_ and ε_*n*_ at [−1, 1], we set no bound constraints on ε_*p*_ and ε_*n*_. We believe such boundary limits are useful for practical purposes in MLE but not in HBA methods. For those parameters with no bound constraints:

(9) $$\begin{array}{l} \xi \sim \text{Normal}(\mu_{\xi },\sigma_{\xi }),\mu_{\xi }\sim \text{Normal}(0,5),\\ \sigma_{\xi }\sim \text{Uniform}(0,1.5) \end{array}$$

For parameters that are constrained to be greater than zero but with an upper limit (= *U*) (e.g., *U* = 2 for α, *U* = 10 for λ, *U* = 5 for *c*), we used the following transformations to allow a flat prior distribution over a full range:

(10) $$\begin{array}{l} \mu_{\xi^{\prime }}\sim \text{Normal}(0,1),\sigma_{\xi^{\prime }}\sim \text{Uniform}(0,1.5),\\ \xi^{\prime }\sim \text{Normal}(\mu_{\xi^{\prime }},\sigma_{\xi^{\prime }}),\xi =U\cdot \text{Probit}(\xi^{\prime }) \end{array}$$

We also reparameterized parameters (i.e., parameters are sampled as independent unit normals and then transformed accordingly for each parameter), which can be effective for complex hierarchical models, as suggested by Stan developers (see Chapter 19 “Optimizing Stan Code” of the Stan 2.1.0 Manual; https://github.com/stan-dev/stan/releases/download/v2.1.0/stan-reference-2.1.0.pdf).

A total of 2000 samples were drawn after 1000 burn-in samples for each of 3 chains (= 2000 samples × 3 chains = a total of 6000 samples). We estimated individual and group parameters separately for each population (HC, amphetamine, and heroin groups). For each parameter, the Gelman-Rubin test (Gelman and Rubin, 1992) was used to check the convergence of the chains (a.k.a. $\hat{R}$ statistic). $\hat{R}$ values close to 1.00 would indicate that MCMC chains are converged to the target distributions. In our data, all model parameters of all models had $\hat{R}$ values of 1.00. MCMC chains were also visually inspected, which confirmed excellent mixing of MCMC samples. Effective sample sizes (ESS) of model parameters, which are related to autocorrelation and mixing of MCMC chains (i.e., a smaller ESS is related to higher autocorrelation), were typically greater than 1000 (out of 6000 total samples). The minimum ESS of hyper-parameters was 561 in the two PVL models, and 372 in the VPP model. Visual inspection of the parameters with smaller ESSs confirmed their convergence to target distributions.

#### Model comparisons using WAIC

WAIC is a way to estimate a model's predictive accuracy with bias correction from over-fitting like Akaike Information Criterion (AIC; Akaike et al., 1973) and Deviance Information Criterion (DIC; Spiegelhalter et al., 2002). As a measure of predictive accuracy, the log predictive density or log-likelihood, log *p*(*y*|θ), is commonly used where *y* and θ indicate data and model parameters, respectively. WAIC is “a more fully Bayesian approach” that uses log pointwise posterior predictive density (*lppd*) and a correction (or penalty) term, each of which can be computed from MCMC samples made available from (hierarchical) Bayesian parameter estimation (for reviews and more details, see Gelman et al., 2013a,b).

Computed lppd (for each participant *i*; subscript *i* is omitted for convenience) is defined as:

(11) $$\sum_{t=1}^{T}\log\left(\frac{1}{S}\sum_{s=1}^{S}p\left(y_{t}|\theta^{s}\right)\right)$$

Here θ^*s*^ are posterior MCMC samples (*s* = 1, 2, …, *S*) and *T* is the number of trials (data points). Note that the likelihood dominates the posterior under standard conditions where a posterior distribution approaches a normal distribution (Degroot, 1970; Gelman et al., 2013a,b).

There is a correction term that adjusts for the effective number of parameters and overfitting. There are two types of adjustments (*p*_*WAIC*1_ and *p*_*WAIC*2_) (Gelman et al., 2013a,b). Gelman et al. (2013a,b) recommended *p*_*WAIC*2_ because of its closer relationship with leave-one-out cross validation than *p*_*WAIC*1_. We report results using *p*_*WAIC*2_ but both adjustments yielded very similar values. Computed *p*_*WAIC*2_ (for each participant *i*, subscript *i* is omitted for convenience here) is defined as:

(12) $$\sum_{t=1}^{T}V_{s=1}^{S}\left(\log p\left(y_{t}|\theta^{s}\right)\right)$$

where *V*^*S*^_*s* = 1_ indicates the sample variance (i.e., the variance of log *p*(*y*_*t*_ |θ ^*s*^) over *S* samples). WAIC_*i*_ for each participant *i* is defined like the following so that its value is on the deviance scale like AIC, DIC, and BIC (Schwartz, 1978).

(13) $${\text{WAIC}}_{i}=-2*(\text{lppd}-p_{WAIC2})$$

We computed lppd and *p*_*WAIC*2_ by rewriting the separate likelihood function in R (R Development Core Team, 2009) but it is also possible to implement WAIC in a Stan code directly (Vehtari and Gelman, under review). Specifically; we first randomly sampled 1,000 (*S* = 1,000 in Equations 11 and 12) posterior samples from each subject's individual posterior distributions. We used posterior individual distributions (instead of group distributions) for the calculation because our goal was to replicate new data and evaluate predictive accuracy in existing groups. Then we prepared a matrix of each subject for trial-by-trial predictive density (*p*(*y*_*t*_ | θ ^*s*^), matrix size = number of samples × number of trials = 1000 × 100). Trial-by-trial predictive density was computed for each subject using each posterior sample separately. Then, using Equations (11–13), we computed lppd, *p*_*WAIC*2_, and WAIC_*i*_ for each participant, and then summed WAIC_*i*_ over all participants for each model (**Table 3**). The R codes for performing HBA and computing WAIC are available by request to the first author (Woo-Young Ahn; wooyoung.ahn@gmail.com).

#### Simulation method

We also used a simulation method to evaluate how accurately a model can generate observed choice pattern in new and unobserved payoff sequences based on parameter values alone (Ahn et al., 2008; Fridberg et al., 2010; Steingroever et al., 2013, 2014). Using the procedure in Appendix B of Ahn et al. (2008) and individual posterior means as a subject's best fitting parameters, we tested the simulation performance of each model. We set the maximum number of trials to 100 and used the payoff schedule of the modified IGT. We only report the results using individual posterior means but we note that running simulations using random draws from individual posteriors (Steingroever et al., 2013, 2014) yielded very similar results (not reported for brevity).

#### Parameter recovery tests

Using parameter recovery tests, we tested the adequacy of each model, specifically how well each model can recover true parameter values that were used to simulate synthetic data (Ahn et al., 2011; Steingroever et al., 2013). We simulated HC participants' performance on the modified IGT assuming that they behaved according to each model. We generated true parameter values based on the individual posterior means of the HC group. Then we simulated synthetic behavioral data based on the parameters, and then recovered their parameter values using the HBA described in Section Hierarchical Bayesian Parameter Estimation. See Appendix for the details.

#### Hierarchical Bayesian multiple regression analyses

For multiple regression analyses, often many candidate predictors are included in the model, which increases the risk of erroneously deciding that a regression coefficient is non-zero. In many cases, regression coefficients are distributed like a *t* distribution, such that the predicted variable has non-significant correlations with most candidate predictors, but a sizable relationship with only a few predictors. Also, some predictors are substantially correlated with each other, which suggests that estimating regression coefficients separately for each predictor can possibly be misleading.

We assigned a higher-level distribution across the regression coefficients of the various predictors. Specifically, regression coefficients came from a *t* distribution with parameters (mean, scale, and df) estimated from the data. Because of this hierarchical structure, estimated regression coefficients experience shrinkage and are less likely to produce false alarms. We used the program “MultiLinRegressHyperJAGS.R” from Kruschke (2011b), available at http://www.indiana.edu/%7Ekruschke/DoingBayesianDataAnalysis/Programs/.

We used Just Another Gibbs Sampler (JAGS) for MCMC sampling and for posterior inference of regression analyses. For each analysis, a total of 50,000 samples per chain were drawn after 1000 adaptive and 1000 burn-in samples with three chains. For each parameter, the Gelman-Rubin test was run to confirm the convergence of the chains. $\hat{R}$ mean values were 1.00 for all parameters.

#### Bayesian estimation for group comparisons

**Figure 1:**
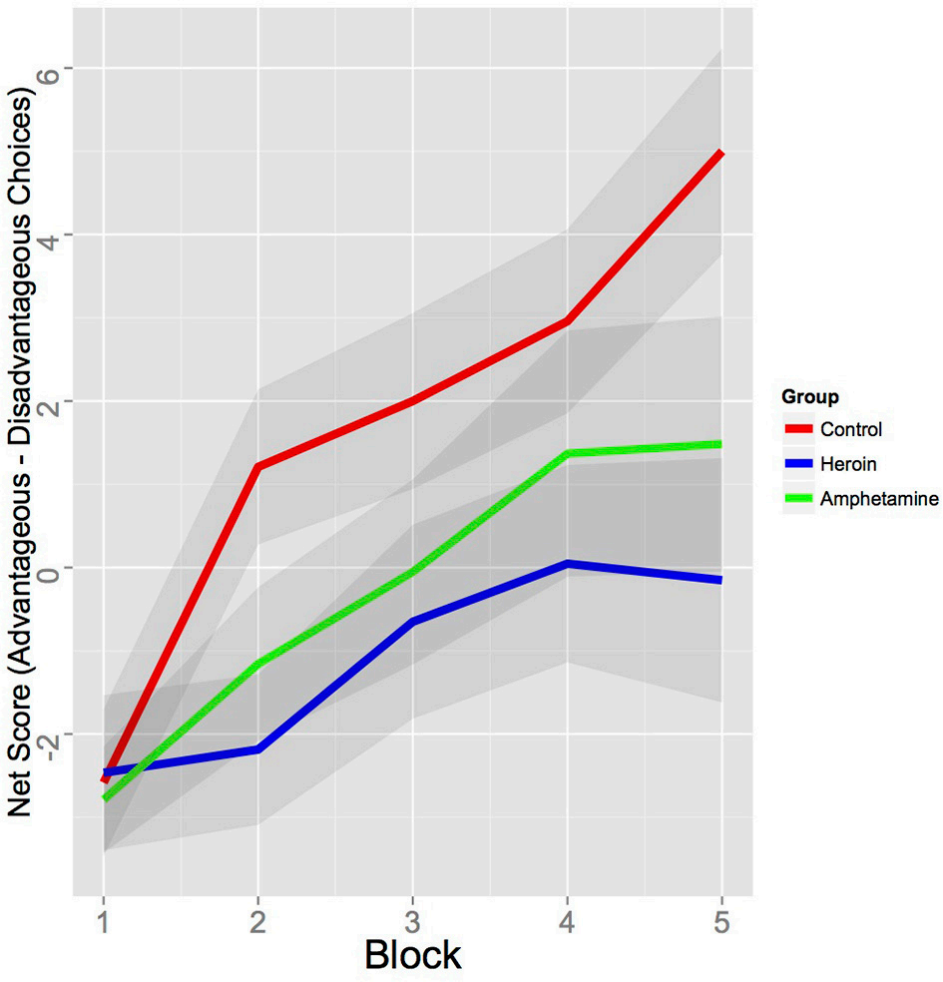
**Behavioral performance on the IGT (net score of “advantageous”—“disadvantageous” choices) of amphetamine, heroin, and healthy control groups**. The 100 trials were divided into five blocks of 20 trials. Shaded regions indicate ±1 s.e.m.

For Bayesian estimation for group differences, (e.g., on behavioral performance, Figure 1), we used Bayesian estimation (BEST) codes that are available at: http://www.indiana.edu/~kruschke/BEST/. The analysis is implemented in JAGS and we used a total of 50,000 samples after 1000 adaptive and 1000 burn-in samples were drawn. $\hat{R}$ mean values were 1.00 for all parameters. For more details about BEST, see Kruschke (2013).

## Results

### Participants' characteristics

Table 1 shows demographic and substance use characteristics of participants. The groups differed on age, such that HC individuals were younger than heroin users [95% HDI from 3.5 to 6.8, mean of HDI = 5.1; *t*_(89)_ = 4.81, *p* = 6.11E-06] and older than amphetamine users [95% HDI from 0.1 to 3.4, mean of HDI = 1.8; *t*_(84)_ = 2.11, *p* = 0.037], reflecting the timeline of heroin and amphetamine influx in Bulgaria. HC individuals had higher IQ than both amphetamine [95% HDI from 0.4 to 11.1, mean of HDI = 6.0; *t*_(84)_ = 2.28, *p* = 0.025] and heroin users [95% HDI from 2.9 to 12.8, mean of HDI = 7.8; *t*_(89)_ = 3.13, *p* = 0.002], but there was no difference between the two drug-using groups [95% HDI from −7.8 to 3.6; mean of HDI = −2.0; *t*_(79)_ = 0.66, *p* = 0.510].

**Table 1:** **Demographic and substance use characteristics of participants**.

	**Healthy control (HC) (***N*** = **48**)**	**Amphetamine (A) (***N*** = **38**)**	**Heroin (H) (***N*** = **43**)**	**Sig.^f^**
Age^a^	24.7 (4.9)	22.7 (3.7)	29.7 (5.0)	*p* < 0.001
Gender (%male)	79.2	76.3	81.4	*p* = 0.85
IQ^b^	112.5 (11.3)	106.7 (12.5)	104.9 (11.9)	*p* = 0.007
Education (years)^c^	13.8 (2.2)	12.5 (1.7)	13.3 (2.5)	*p* < 0.001
Years of amph./heroin use	–	3.2 (2.3)	7.2 (3.5)	*p* < 0.001
Years of any drug use	–	6.5 (2.7)	10.8 (3.6)	*p* < 0.001
# of amph./heroin DSM-IV dependence criteria met	–	4.9 (1.3)	6.1 (1.0)	*p* < 0.001
Time (years) since last met dependence criteria	–	2.8 (1.6)	2.9 (2.2)	*p* = 0.89
Fagerstrom test of nicotine dependence^d^	0.7 (1.6)	3.3 (2.8)	4.7 (2.7)	*p* < 0.001
Min–Max days since last drug use	–	90–2190	152–3285	–
Past cannabis dependence (%)^e^	0	12 (32%)	6 (14%)	*p* < 0.001

a *H > HC > A (Bayesian and NHST t-tests yielded the same conclusions)*.

b *HC > A, H (Bayesian and NHST t-tests yielded same conclusions)*.

c *HC > A (Bayesian and NHST t-tests yielded same conclusions)*.

d *H > A > HC (Bayesian and NHST t-tests yielded same conclusions)*.

e *A > H > HC (Bayesian and NHST χ-square tests yielded same conclusions)*.

f *Sig. results are based on omnibus NHST ANOVA tests*.

As reported in Table 2, the two drug using groups scored higher on trait impulsivity (BIS-11) [HC vs. Amphetamine: 95% HDI from 5.5 to 14.9, mean of HDI = 10.2; *t*_(83)_ = 4.66, *p* = 1.19E-05; HC vs. Heroin: 95% HDI from 5.6 to 13.7, mean of HDI = 9.7; *t*_(88)_ = 4.87, *p* = 4.90E-06] and psychopathy (PCL:SV) [HC vs. Amphetamine: 95% HDI from 4.0 to 7.7, mean of HDI = 5.8; *t*_(84)_ = 6.49, *p* = 5.72E-09; HC vs. Heroin: 95% HDI from 7.4 to 11.1, mean of HDI = 9.3; *t*_(89)_ = 10.62, *p* = 2.20E-16] than HC individuals. Comparisons between the two drug using groups revealed that heroin users had higher levels of psychopathy than amphetamine users [HDI from 0.8 to 5.1, mean of HDI = 3.0; *t*_(79)_ = 2.73, *p* = 0.008]. Both amphetamine and heroin users scored higher on depression (BDI-II) [HC vs. Amphetamine: 95% HDI from −4.4 to −0.5, mean of HDI = −2.3; *t*_(82)_ = 2.26, *p* = 0.026; HC vs. Heroin: 95% HDI from −5.8 to −1.7, mean of HDI = −3.8; *t*_(88)_ = 3.59, *p* = 5.40E-04], state anxiety (STAI-S) [HC vs. Amphetamine: 95% HDI from −7.7 to −1.6, mean of HDI = −4.5; *t*_(84)_ = 2.90, *p* = 4.7E-04; HC vs. Heroin: 95% HDI from −9.7 to −2.5, mean of HDI = −6.4; *t*_(89)_ = 3.90, *p* = 1.80E-04], and trait anxiety (STAI-T) [HC vs. Amphetamine: 95% HDI from −8.5 to −0.3, mean of HDI = −4.4; *t*_(84)_ = 2.18, *p* = 0.032; HC vs. Heroin: 95% HDI from −10.0 to −1.3, mean of HDI = −5.6; *t*_(89)_ = 2.86, *p* = 0.005] than HC individuals. There were no differences between the two drug using groups on these measures.

**Table 2:** **Personality and psychopathology characteristics of participants**.

	**HC**	**A**	**H**	**Group comparisons**
BIS total	55.96 (9.1)	66.13 (11.0)	65.70 (9.9)	HC < A, H
BIS attention	14.28 (3.7)	16.32 (4.1)	16.56 (5.3)	HC < A, H
BIS motor	20.40 (3.8)	25.18 (5.2)	23.12 (5.0)	HC < A, H
BIS nonplanning	21.23 (4.3)	24.63 (4.4)	25.70 (3.9)	HC < A, H
PCL:SV	3.67 (3.2)	9.34 (4.9)	12.19 (4.4)	HC < A < H
BDI-II total	4.21 (4.1)	6.62 (5.6)	8.26 (6.4)	HC < A, H
State anxiety (STAI-S)	29.42 (5.9)	33.68 (7.7)	36.12 (10.1)	HC < A, H
Trait anxiety (STAI-T)	34.33 (8.7)	38.58 (9.3)	39.98 (10.1)	HC < A, H

*All group comparison results are based on Bayesian tests. HC, healthy controls; A, amphetamine; H, heroin; BIS, Barratt Impulsiveness Scale; PCL:SV, Psychopathy Checklist: Screening Version; BDI-II, Beck Depression Inventory-II; STAI, State Trait Anxiety Inventory*.

### Behavioral results

Behavioral results revealed that the HC group made more advantageous choices than the heroin group [difference of mean net score (advantageous—disadvantageous choices per five blocks of 20 trials) = 2.77, 95% HDI from 0.7 to 4.8, mean of HDI = 2.8; *t*_(90)_ = 2.80, *p* < 0.010] and marginally than the amphetamine group [difference of mean net score = 1.14, 95% HDI from −0.1 to 2.3, mean of HDI = 1.9; with 95.3% of the posterior samples were greater than 0; *t*_(84)_ = 2.02, *p* = 0.047]. There were no behavioral differences between the two drug using groups in terms of net scores (see Figure 1). Further, the choice patterns of these two groups were qualitatively different from those of the HC group. As shown in Figures S1–S3 (left), whereas the HC group favored one of the advantageous decks (Deck D) as the task progressed, both amphetamine and heroin users consistently favored the disadvantageous deck B throughout the task. Decks B and D carry low-frequency losses and are usually chosen more often than decks with high-frequency losses such as A and C, yet one is disadvantageous (Deck B) whereas the other one is advantageous (Deck D). Our results demonstrate that past drug users who are currently in protracted abstinence continue to show similar preference for disadvantageous decks as currently dependent drug users (Bechara et al., 2001; Yechiam et al., 2005).

### Model comparisons results

We first checked which model provided the best predictive accuracy, as measured by WAIC. Table 3 presents WAIC scores for each model, summarized for each group. Note that the smaller a model's values of WAIC scores are, the better its model-fits are. As noted in Table 3, the VPP model provided the best model-fits relative to the other models in all groups, followed by the PVL-DecayRI. These results are consistent with previous reports from Worthy et al. (2013b).

**Table 3:** **WAIC scores of each model, computed separately for each group**.

**Model**	**WAIC_**HC**_**	**WAIC_**A**_**	**WAIC_**H**_**	**WAIC_**Sum**_**
VPP	11659.4	9114.7	10168.1	30942.2
PVL-DecayRI	12145.6	9521.0	10752.4	32419.0
PVL-Delta	12448.8	9747.3	11036.4	33232.5

*The best-fitting model in each group is underlined*.

*HC, healthy controls; A, amphetamine; H, heroin*.

The simulation method and parameter recovery tests yielded somewhat different findings (Figures S1–S3). Consistent with previous reports (Ahn et al., 2008; Fridberg et al., 2010; Steingroever et al., 2013, 2014), the PVL-Delta model showed good simulation performance in all three groups, adequately predicting the rank order of four decks and good parameter recovery (Figure A3). The PVL-DecayRI model also captured the global pattern of deck preference in all groups even if it failed to fully capture the preference reversal of certain decks over trials (e.g., decks A and C in the heroin group, Figure S3). Parameter recovery tests yielded somewhat mixed results (Figure A2): A (decay rate) and c (response consistency) were recovered well, but performance on α (reward sensitivity) and λ (loss aversion) were not as good as with the PVL-Delta. The VPP model, on the other hand, showed the worst simulation and parameter recovery performance: the model over-estimated the preference of deck C in the HC and amphetamine groups and failed to predict the preference of deck C over deck A in the heroin group. These results are inconsistent with the simulation results of Worthy et al. (2013b), in which the VPP model showed the best simulation performance. However, HC participants in Worthy et al. (2013b) continued to prefer the disadvantageous deck (Deck B) throughout the task, unlike our HC participants who preferred the advantageous Deck D. Worthy et al. (2013b) reported simulation performance by averaging choice probabilities across all trials in each deck (Figure 2A in Worthy et al., 2013b). If we used the same criterion, the VPP model performs quite well for the heroin group, in which deck B is most strongly preferred and preference for decks A and C are similar on average. Another major difference between our study and Worthy et al. (2013b) is the parameters used for the simulation method: Worthy et al. (2013b) used MLE estimates whereas we used HBA estimates, which may lead to somewhat different simulation performance. With respect to parameter recovery (Figure A1) with the VPP model, posterior distributions of several parameters were very broad (e.g., ω) and some parameters were not well estimated (e.g., *k*), which might be attributed to its higher number of parameters compared to the two PVL models (8 vs. 4).

**Figure 2:**
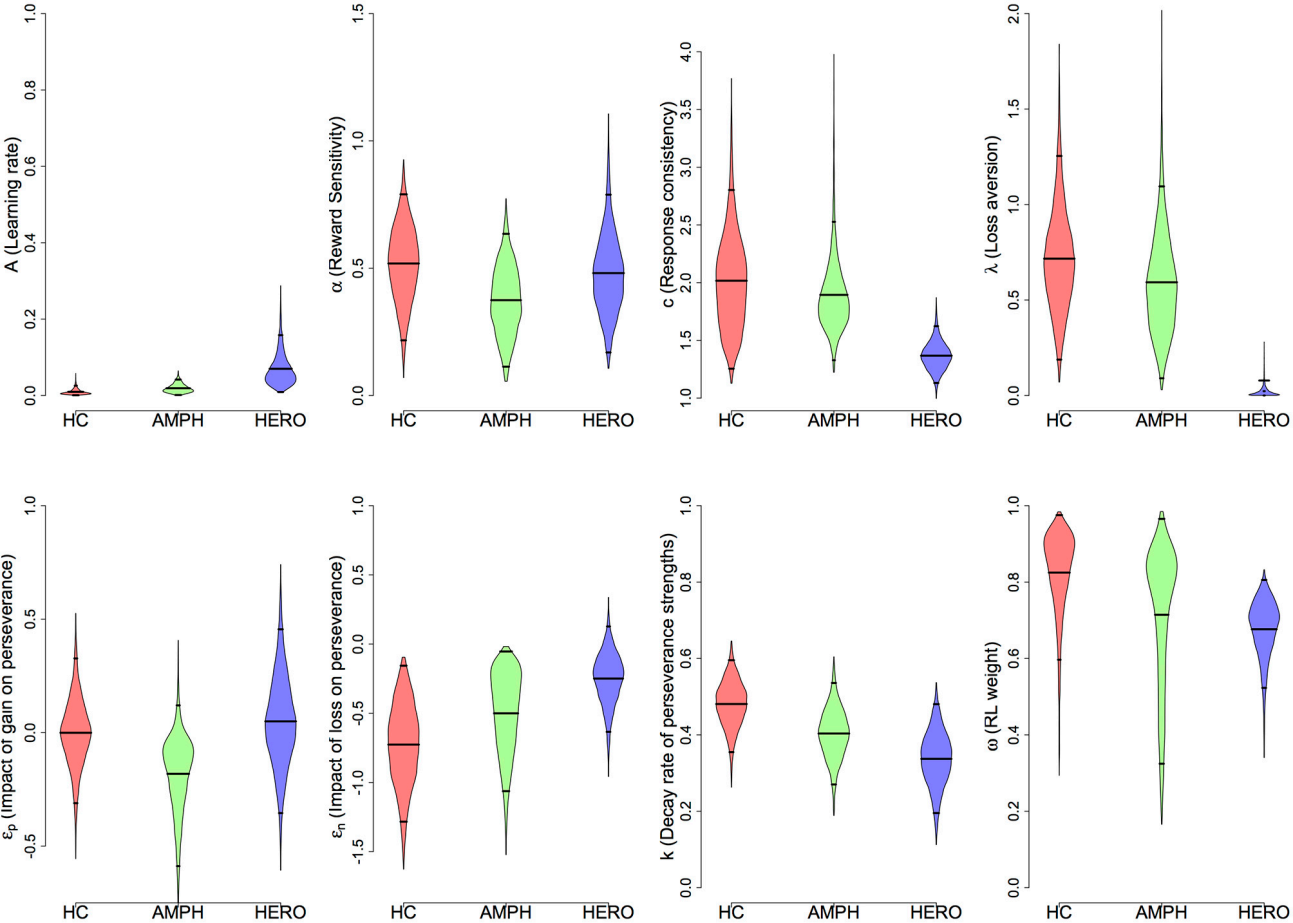
**Density plots of posterior group parameter distributions with the Value-Plus-Perseverance (VPP) model**. Bottom and top tick marks indicate HDI 95% range, and middle tick marks indicate mean values for each group. Density plots range from 0.01 to 99.99% of posterior distributions. HC, Healthy Control group; AMPH, Amphetamine group; HERO, Heroin group.

**Figure 3:**
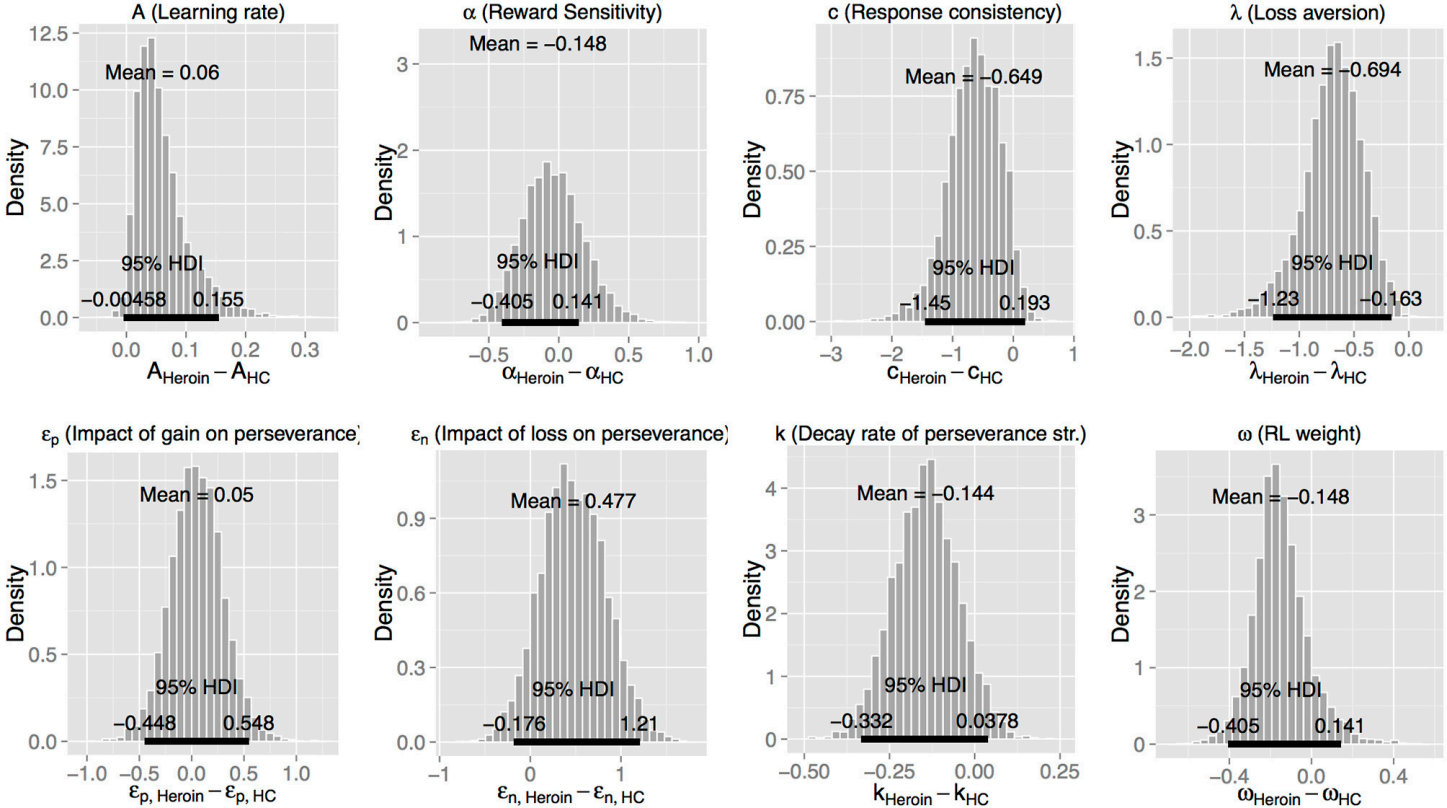
**Posterior distributions of differences of group mean parameters between the heroin and the healthy control (HC) groups, with the VPP model**. HDI, highest density interval.

Next, we used the best-fitting (VPP) model to compare the three groups (Figure 2 and Table 4). Heroin users displayed reduced loss aversion (λ) compared to HC [95% HDI from −1.2 to −0.2, mean of HDI = −0.7; *t*_(89)_ = 8.33, *p* = 9.024E-13] and amphetamine users [95% HDI from 0.1 to 1.1, mean of HDI = 0.6; *t*_(79)_ = 6.82, *p* = 1.63E-09] (see Figure 3 for the 95% HDI of group differences between heroin and HC groups and Figures S4, S5 for the 95% HDI of group differences between amphetamine and other groups). In contrast, our hypothesis that reward sensitivity (α) would be higher in amphetamine users compared to HC was not supported. The learning rate (*A*) was marginally different between the heroin and the HC groups [95% HDI from −0.0 to 0.2, mean of HDI = 0.1; *t*_(89)_ = 4.91, *p* = 4.08E-06, Figure 3].

**Table 4:** **Means and standard deviations (in parentheses) of group mean parameters with the VPP model**.

**VPP parameters**	**HC**	**A**	**H**
Learning rate (*A*)	0.010 (0.008)	0.019 (0.011)	0.070 (0.044)
Reward sensitivity (α)	0.518 (0.149)	0.374 (0.137)	0.481 (0.159)
Response sensitivity (*c*)	2.017 (0.419)	1.894 (0.329)	1.368 (0.125)
Loss aversion (λ)^a^	0.717 (0.273)	0.593 (0.275)	0.023 (0.033)
Perseverance after gain (ε_*p*_)	−0.001 (0.154)	−0.181 (0.179)	0.050 (0.204)
Perseverance after loss (ε_*n*_)	−0.726 (0.296)	−0.500 (0.297)	−0.249 (0.192)
Perseverance decay rate (*k*)	0.481 (0.062)	0.404 (0.067)	0.337 (0.073)
RL weight (ω)	0.825 (0.110)	0.714 (0.183)	0.677 (0.078)

*HC, healthy controls; A, amphetamine; H, heroin*.

a *HC, A > H*.

We further checked whether the group differences we found using the best-fitting (VPP) model are consistent when tested with other models (PVL-DecayRI and PVL-Delta). Tables 5, 6 summarize the mean group parameter estimates of the PVL-DecayRI (see Figures S6–S8 for the 95% HDI of group differences) and PVL-Delta (see Figures S9–S11 for the 95% HDI of group differences), respectively. As seen in Figures 3, S6, and S9, we consistently found reduced loss aversion in heroin users compared to HC, whichever model we used. The PVL-DecayRI model showed increased reward sensitivity (α parameter) in amphetamine users compared to HC [Figure S7, 95% HDI from 0.0 to 0.5, mean of HDI = 0.3; *t*_(84)_ = 6.26, *p* = 1.53E-08], which was not replicated with other models.

**Table 5:** **Means and standard deviations (in parentheses) of group mean parameters with the PVL-DecayRI model**.

**PVL DecayRI parameters**	**HC**	**A**	**H**
Decay rate (*A*)	0.736 (0.068)	0.809 (0.072)	0.730 (0.087)
Reward sensitivity (α)^a^	0.053 (0.043)	0.310 (0.129)	0.122 (0.074)
Response sensitivity (*c*)	0.216 (0.038)	0.186 (0.040)	0.210 (0.050)
Loss aversion (λ)^b^	1.262 (0.543)	0.910 (0.494)	0.110 (0.108)

*HC, healthy controls; A, amphetamine; H, heroin*.

a *HC < A*.

b *HC > H*.

**Table 6:** **Means and standard deviations (in parentheses) of group mean parameters with the PVL-Delta model**.

**PVL Delta parameters**	**HC**	**A**	**H**
Learning rate (*A*)	0.037 (0.019)	0.035 (0.018)	0.172 (0.080)
Reward sensitivity (α)	0.382 (0.126)	0.283 (0.116)	0.475 (0.123)
Response sensitivity (*c*)	1.285 (0.204)	1.292 (0.181)	0.947 (0.147)
Loss aversion (λ)^a^	0.650 (0.240)	0.376 (0.220)	0.060 (0.055)

*HC, healthy controls; A, amphetamine; H, heroin*.

a *HC > H*.

Given that the groups differed on age, IQ, and education, we conducted NHST Analysis of Covariance (ANCOVA) tests to examine whether group differences on model parameters remain significant after controlling for these factors. Dependent variables were model parameter values (individual posterior means), group membership (e.g., HC vs. amphetamine groups) was the categorical independent variable, and covariates were age, IQ, and education. With any model (VPP, PVL-DecayRI, or PVL-Delta), group difference on loss aversion between heroin and HC groups remained significant [e.g., with the VPP model, *F*_(1, 86)_ = 26.06, *p* = 1.16E-13]. The group difference on reward sensitivity between amphetamine and HC groups with the PVL-DecayRI model also remained significant [*F*_(1, 81)_ = 46.28, *p* = 1.61E-09].

### Exploratory analyses: associations of model parameters with substance use and personality characteristics

Next, we examined associations of model parameters of the impaired neurocognitive processes (loss aversion for heroin users using the VPP model) with substance use characteristics (number of years of drug use, length of abstinence from primary drug, number of DSM-IV criteria met for primary drug of dependence, nicotine dependence, and past cannabis dependence), impulsive personality traits (BIS-11) and impulse-related personality disorders (PCL:SV). As noted earlier, we used hierarchical robust Bayesian multiple linear regression, which has a hyperdistribution on regression coefficients across predictors and large-tail distributions to accommodate outliers. The results showed that loss aversion in heroin users was not predicted by any variable (Figure S12 for the robust Bayesian multiple linear regression results). None of the regressors were significant (*p* < 0.05 with NHST).

In contrast to our null findings with the VPP model, we found two associations when we used the affected parameters from the PVL-DecayRI model (loss aversion for heroin users and reward sensitivity for amphetamine users). In heroin users, loss aversion (λ) was predicted by impulsive personality traits (BIS-11 total score; mean coefficient = −0.027, 95% HDI from −0.05 to −0.00, mean of HDI = −0.03) (Figure S13). In contrast, in amphetamine users, reward sensitivity was predicted by number of years of drug use (mean coefficient = 0.042, 95% HDI of group differences from 0.01 to 0.07, mean of HDI = 0.04, see Figure S14). Other variables were not associated with model parameters. Correlational analyses with internalizing characteristics (depression and anxiety) revealed no associations with model parameters.

## Discussion

This is the first human study that uses a computational modeling approach to investigate neurocognitive functioning in relatively pure amphetamine and heroin users. Our behavioral results reveal that heroin users show more disadvantageous decision-making performance than HC; however, their performance was not different from that of amphetamine users. These results are in line with the persistent nature of decision-making deficits observed among opiate addicts in particular (Vassileva et al., 2007b; Fernández-Serrano et al., 2011; Li et al., 2013). Critically, our computational modeling findings suggest that amphetamine and heroin users may be characterized by dissociable decision-making biases even within the context of no overt behavioral differences in performance. When we compared groups using the best-fitting (VPP) model, heroin users showed reduced loss aversion relative to amphetamine users and HC. Notably, the reduced loss aversion among heroin users compared to healthy individuals was robust across all models we tested. With regards to amphetamine users, we did not find any distinct decision-making profile using the best-fitting VPP model. However, when using the PVL-DecayRI model, which had the second best model-fits in our data, amphetamine users showed greater reward sensitivity than HC. These group differences were at the outcome evaluation stage according to a recent framework of value-based decision-making (Rangel et al., 2008) and putatively reflect an emotional and activation type of self-regulation (Bickel et al., 2012).

We tested three existing cognitive models to compare the two drug user groups with HC. Consistent with previous reports (Worthy et al., 2013b), we found that the VPP model was the best-fitting model when measured by WAIC, followed by the PVL-DecayRI and the PVL-Delta. However, it should be noted that the VPP model has twice as many parameters as other models (8 vs. 4) and showed the worst simulation and parameter recovery performance compared to the two PVL models. In contrast, Worthy et al. (2013b) show good simulation performance for the VPP model in their dataset; however, there are two major differences between their study and ours. First, in Worthy et al. (2013b), control participants preferred the disadvantageous deck (Deck B) throughout the task, similar to the amphetamine and heroin groups in our study. Indeed, the simulation performance of the VPP model is quite good for the heroin group if we collapse trial-by-trial simulation performance over trials on each deck. Second, Worthy et al. (2013b) used MLE estimates instead of HBA estimates. Thus, it remains to be determined whether the poor simulation performance of the VPP model in our datasets is due to its over-complexity, the limited generalizability of specific behavioral patterns, or to differences in the parameter estimation methods. It would also be helpful to perform external validation tests (e.g., Wallsten et al., 2005) because the parameters of a model with good model-fits do not necessarily reflect underlying psychological constructs (Riefer et al., 2002). In this study, each participant performed only up to 100 trials: Even if hierarchical modeling allowed us to pool information across individuals, 100 trials might not contain enough information to reliably estimate 8 free parameters and capture true underlying psychological constructs. It might be related to the fact that behaviorally the amphetamine group showed different choice patterns from the HC group but none of their model parameter values are credibly different from those of the HC group. As seen in Figure 2, several parameters of the amphetamine group are “sub-optimal” compared to the HC group (e.g., ε_*n*_, *k*, and ω) but the group differences did not reach the threshold of credible group difference. It is possible that deficits in the amphetamine group were decomposed into several parameters, instead of into one or two parameters in the VPP model. It may be necessary and helpful to develop new models with fewer model parameters based on the psychological and neuroscience literature by using model comparison methods and performing external validation.

There are a few previous studies using the PVL-DecayRI (Vassileva et al., 2013) or the PVL-Delta (Fridberg et al., 2010) model to study decision-making processes in drug users. Consistent with our results, both chronic (current) marijuana users (Fridberg et al., 2010) and polysubstance (former) users (Vassileva et al., 2013) showed reduced loss aversion compared to HC. On the other hand, chronic marijuana users also exhibited higher reward sensitivity, impaired learning/memory, and reduced response consistency compared to HC when tested with the PVL-Delta model (Fridberg et al., 2010). Polysubstance use was also associated with impaired learning/memory when tested with the PVL-DecayRI model (Vassileva et al., 2013). Stout et al. (2004) used the EVL model and MLE method for parameter estimation, and reported reduced attention weight to loss among current cocaine users compared to HC. In the EVL model, the *w* parameter (attention weight to loss vs. gain) incorporates both reward sensitivity and loss aversion; therefore, it is difficult to directly compare the findings from Stout et al. (2004) with our results. However, it is likely that one or both of the two processes was impaired in current cocaine users in the Stout et al. (2004) study.

It should be also noted that the mean *w* parameter (RL weight) value was greater than 0.5 in all groups (Figure 2), suggesting that overall RL was a primary strategy in all groups. Worthy et al. (2013b) reported that the mean *w* parameter of healthy individuals was 0.49, which is the mean value of MLE individual estimates. In addition to the difference in parameter estimation methods, we also found some differences in the choice patterns of the three groups. As seen in Figure S1, healthy control individuals in our study eventually preferred the advantageous deck (Deck D) as the task progressed. On the other hand, healthy individuals in Worthy et al. (2013b) continued to prefer the disadvantageous deck (Deck B) throughout the task, which was the deck preferred by both heroin and amphetamine users in our study. It remains unclear why the two drug user groups, which showed similar behavioral patterns to participants in Worthy et al. (2013b), showed *w* value greater than 0.5 on average. A future study will be necessary to replicate the findings.

This is one of the very few studies that investigate amphetamine and heroin users in protracted abstinence (Ersche et al., 2005a,b; Clark et al., 2006). Our results indicate that decision-making deficits previously reported with current drug users (Bechara et al., 2001; Yechiam et al., 2005) may persist long after discontinuation of drug use and appear particularly pronounced in heroin users. These deficits and decision-making biases may have existed prior to onset of drug use and thereby could have contributed to an increased susceptibility to develop addiction, in line with longitudinal studies with adolescents, which show that poor response inhibition and behavioral dysfunction often precede onset of drug use and contribute to the development of addiction (Nigg et al., 2006; Wong et al., 2006). Alternatively, these deficits and biases may reflect residual, enduring and possibly irreversible effects of chronic drug use; or an interaction between pre-existing predispositions and residual effects of drugs of abuse. Although our study revealed some dissociable decision-making biases in amphetamine and heroin users, our design does not allow us to determine whether they precede onset of drug use or whether they are consequences of chronic drug use. This crucial question should be investigated by future carefully designed prospective studies.

Using the second best-fitting PVL-DecayRI model, we found that the distinct decision-making style of heroin users characterized by reduced sensitivity to loss is associated with elevated trait impulsivity, as hypothesized. These findings are in line with reports that personality variables are related to decision-making performance on the IGT among heroin users on OST (Lemenager et al., 2011). Our results indicate that similar associations are observable among heroin users in protracted abstinence who are not on OST. Speculatively, given the persistent nature of personality traits such as impulsivity, which develop early and typically prior to onset of substance dependence, the reduced loss aversion in heroin users may have predated the development of addiction and may be of etiological significance for addiction to opiates in particular. In contrast, the decision-making bias displayed by stimulant users (reward sensitivity) was not associated with personality traits but was instead related to duration of stimulant use, which suggests that such biases may potentially reflect cumulative residual effects of chronic stimulant use. It is important to emphasize that we should exercise caution when interpreting these associations, as they were not replicated with the best-fitting (VPP) model.

A question arises as to what is the clinical significance of the observed decision-making biases and deficits within the context of our participants' history of protracted abstinence, which is the standard metric of success of most addiction treatment programs. Specifically, despite the observed decision-making deficits and biases among the two drug user groups, the majority of our participants have been remarkably successful in maintaining abstinence for long periods of time and without the help of any substitution therapy. In essence, the ability of our participants to abstain for such protracted periods of time suggests that this group could be comprised of some of the least impulsive SDI, expected to display more adaptive decision-making abilities than SDI who are unable to remain abstinent for long. Future studies should determine the real-life significance of such decision-making deficits and biases and the role they play in the protracted abstinence stage. For example, we recently reported that some decision-making biases may have functional significance for HIV infected women with a history of illicit drug use, among whom they may be related to risky sexual behaviors and reduced adherence to HIV medication dosing schedules (Vassileva et al., 2013). Similarly, we recently found that a composite neurocognitive index of reward-based decision-making (which includes the IGT) predicts recent (past 30-days) sexual HIV risk behaviors in heroin and amphetamine users in protracted abstinence (Wilson et al., under review). Overall, our results suggest that decision-making processes other than the ones we examined may be more relevant for the successful and prolonged maintenance of a state of abstinence. Further, our findings may be specific to decision-making under uncertainty and ambiguity, as measured by the IGT. It is possible that SDI in protracted abstinence may display intact functioning in other aspects of decision-making (e.g., decisions under risk) that may have more direct relevance to the successful maintenance of abstinence. On the other hand, the fact that such decision-making deficits and biases were observed in participants who have successfully maintained prolonged abstinence raises the question of whether users who are unable to maintain long-term abstinence are characterized by even more aberrant decision-making profiles. It would be crucial for future studies to determine how “successful” long-term abstainers such as our participants compare to currently active SDI or to SDI who are unable to abstain from drug use. Future studies should also determine whether similar substance-specific biases are observable in opiate and stimulant users at other stages of the addiction cycle and ideally employ longitudinal designs to determine whether they are precursors or consequences of chronic substance use.

While clearly of theoretical significance, the extent to which our findings have implications for prevention and intervention remains to be determined. If replicated by future studies, such decision-making deficits and biases may inform treatment and recovery programs for opiate and stimulant dependent individuals. Within this context, pre-treatment decision-making assessments may represent a useful adjunct to help formulate personalized treatment plans (Baldacchino et al., 2012), which could potentially include cognitive enhancement or training that have shown some promising results (Nutt et al., 2007; Bickel et al., 2011). Our results from the PVL-DecayRI model suggest that interventions that target reduced loss aversion (punishment sensitivity) may be more suitable for heroin users, whereas others addressing increased reward sensitivity may hold promise with amphetamine users, though we should exercise caution with the latter, which failed to replicate with the best-fitting model.

There are a number of limitations that need to be considered when evaluating the current findings. First, the fact that our participants were predominantly male should be taken into account when considering the generalizability of our findings to females. Second, our findings could have been influenced by group differences in age, IQ, and education, though the reduced loss aversion in heroin users and the increased reward sensitivity in the amphetamine group (with the PVL-DecayRI model) relative to HC remained robust even after controlling for those factors. Third, computational modeling parameter estimates, like many conceptual or quantitative interpretive tools, are useful heuristics in the evaluation of observed behavior patterns, not explanatory mechanisms of the phenomena at hand. Interpretations should be rendered accordingly, though the reduced loss aversion in heroin users was robust across all models we tested.

In sum, by recruiting relatively pure amphetamine and heroin users in protracted abstinence and by parcellating their decision-making performance into distinct neurocognitive processes by using computational modeling and Bayesian tools, we revealed that heroin users displayed reduced loss aversion relative to HC while being in protracted abstinence. Future studies utilizing other experimental paradigms probing different aspects of decision-making and computational models will be necessary to examine which mechanisms may be at play in the decision-making performance of heroin and amphetamine users at different stages of the addiction cycle.

### Conflict of interest statement

The authors declare that the research was conducted in the absence of any commercial or financial relationships that could be construed as a potential conflict of interest.
