# Supplementary material for: Decision-making in stimulant and opiate addicts in protracted abstinence: evidence from computational modeling with pure users
Source: Front Psychol. 2014 Aug 12;5:849. doi: 10.3389/fpsyg.2014.00849 (PMC4129374; doi:10.3389/fpsyg.2014.00849)
Supplement: Supplementary file 1 [file DataSheet1.PDF]

Supplementary Material

Decision-making in stimulant and opiate addicts in protracted abstinence: evidence from computational modeling with pure users

Woo-Young Ahn[1], Ph.D., Georgi Vasilev[2], MD, MPH, Sung Ha Lee[3], M.S., Jerome R. Busemeyer[3], Ph.D., John K. Kruschke[3], Ph.D., Antoine Bechara[4,5], Ph.D., and Jasmin Vassileva[6*], Ph.D.

[1]Virginia Tech Carilion Research Institute, Roanoke, VA, USA
[2]Bulgarian Addictions Institute, Sofia, Bulgaria
[3]Department of Psychological and Brain Sciences, Indiana University, Bloomington, IN, USA
[4]Department of Psychology, University of Southern California, Los Angeles, CA, USA
[5]Brain and Creativity Institute, University of Southern California, Los Angeles, CA, USA
[6]Department of Psychiatry, Virginia Commonwealth University School of Medicine, Richmond, VA, USA

*** Correspondence:**
Jasmin Vassileva
Department of Psychiatry, Virginia Commonwealth University, Institute for Drug and Alcohol Studies, 203 E. Cary Street, Richmond, VA 23219, USA
email: jlvassileva@vcu.edu

Keywords: addiction, decision-making, computational modeling, heroin, amphetamines, protracted abstinence, Bayesian data analysis, Widely Applicable Information Criterion (WAIC)

Figures S1-S14

Appendix

Figures A1-A3

Supplementary References

Figure S1. Proportion of choices from each deck of the healthy control (HC) group (each block consists of 20 trials) and simulation performance of each model.

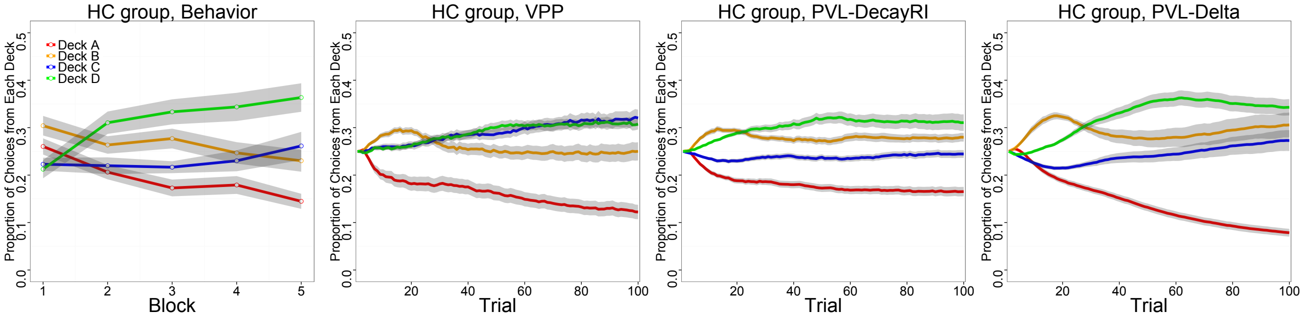

Figure S2. Proportion of choices from each deck of the amphetamine group (each block consists of 20 trials) and simulation performance of each model.

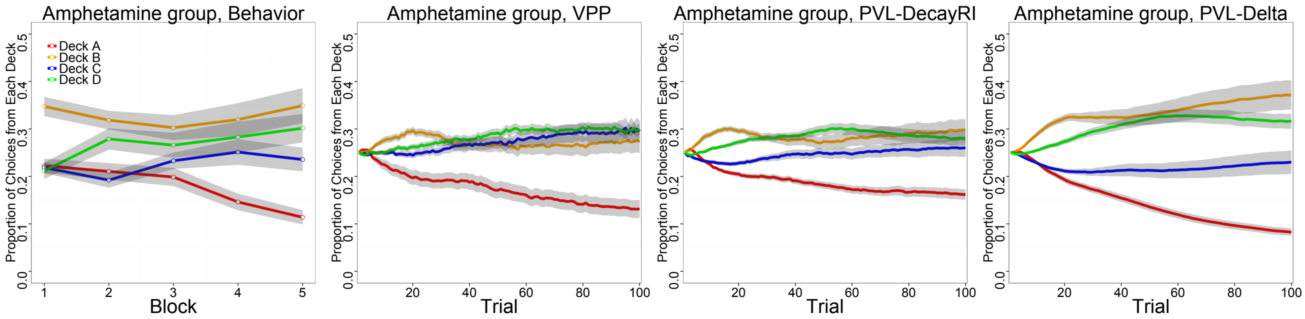

Figure S3. Proportion of choices from each deck of the heroin group (each block consists of 20 trials) and simulation performance of each model.

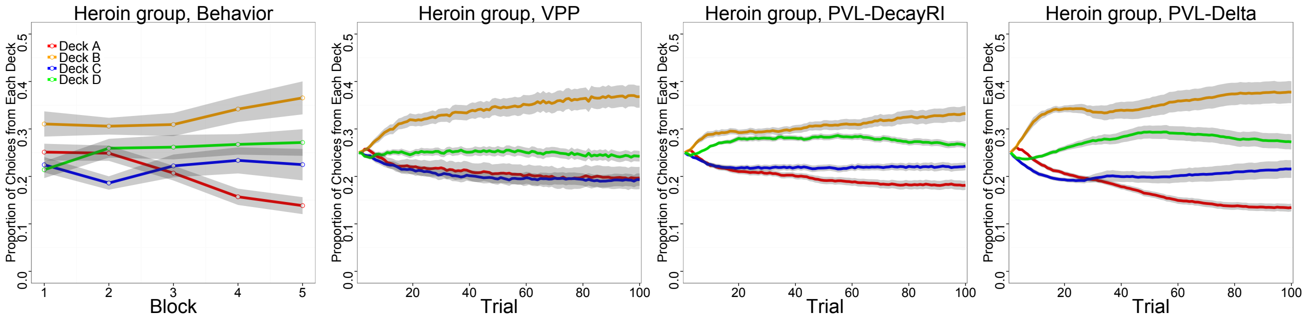

Figure S4. Posterior distributions of differences of group mean parameters between the amphetamine and the healthy control (HC) groups, with the VPP model. HDI = highest density interval.

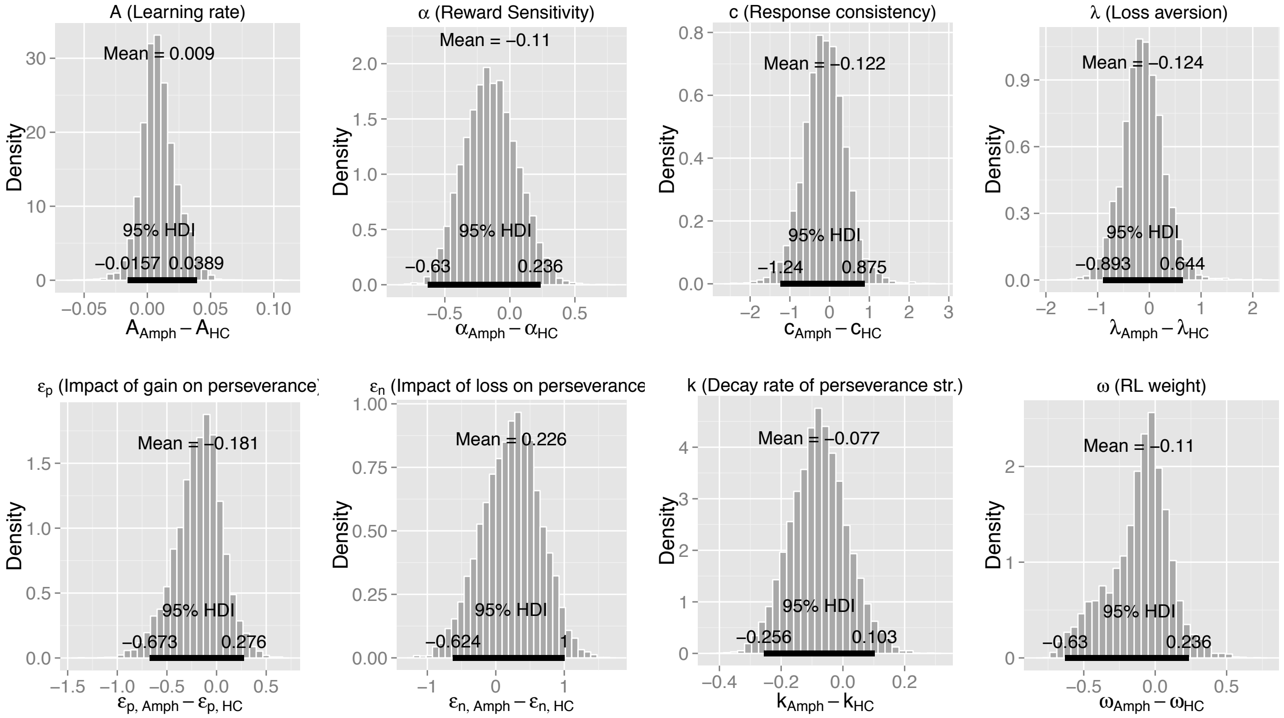

Figure S5. Posterior distributions of differences of group mean parameters between the amphetamine and the heroin groups, with the VPP model. HDI = highest density interval.

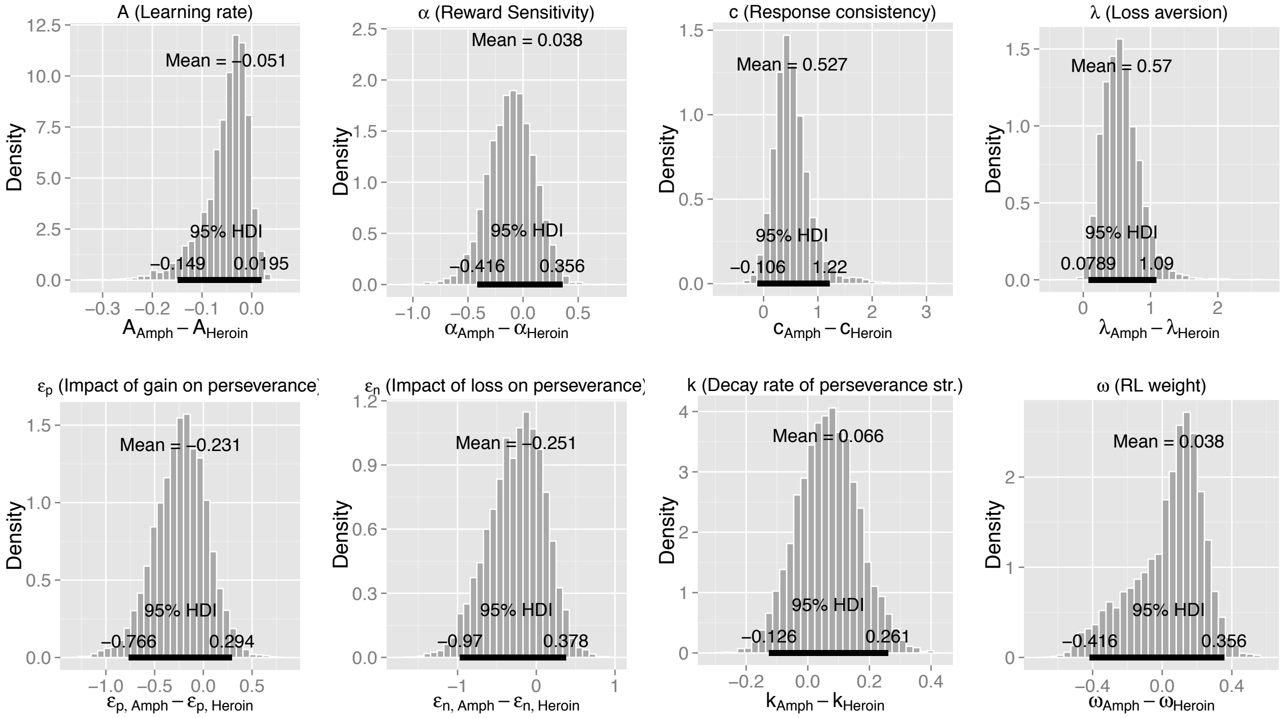

Figure S6. Posterior distributions of differences of group mean parameters between the heroin and the healthy control (HC) groups, with the PVL-DecayRI model. HDI = highest density interval.

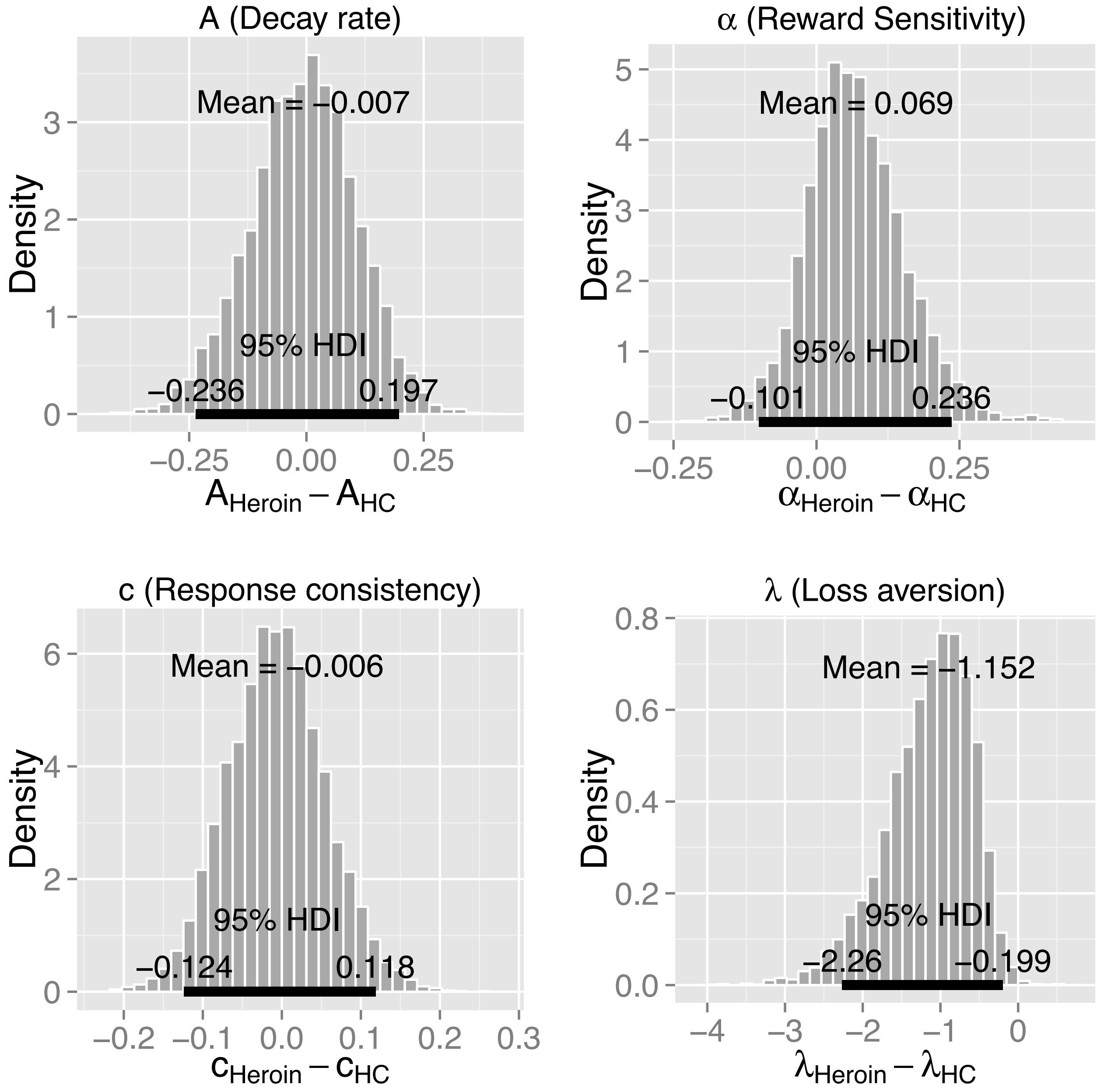

Figure S7. Posterior distributions of differences of group mean parameters between the amphetamine and the healthy control (HC) groups, with the PVL-DecayRI model. HDI = highest density interval.

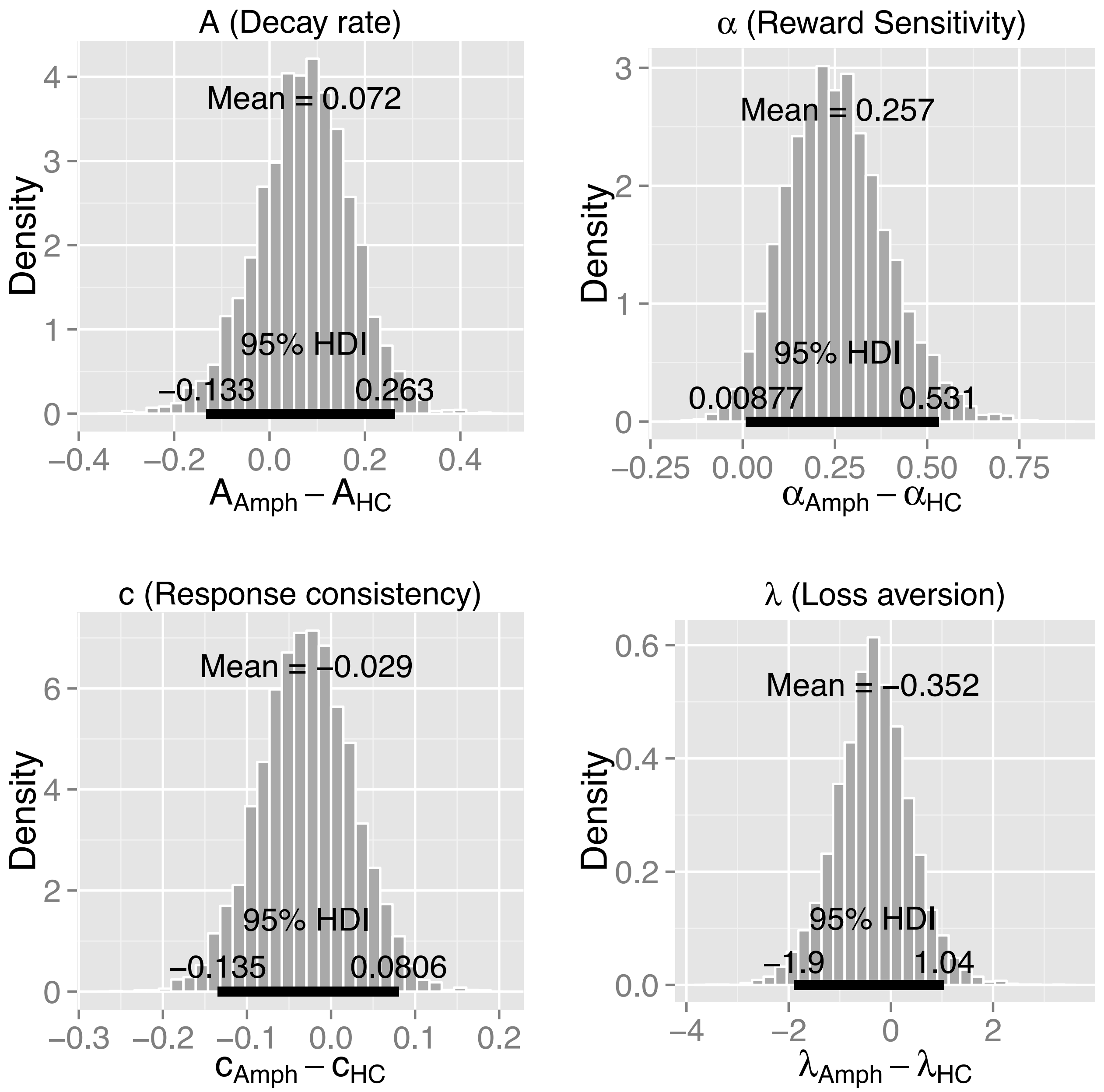

Figure S8. Posterior distributions of differences of group mean parameters between the amphetamine and the heroin groups, with the PVL-DecayRI model. HDI = highest density interval.

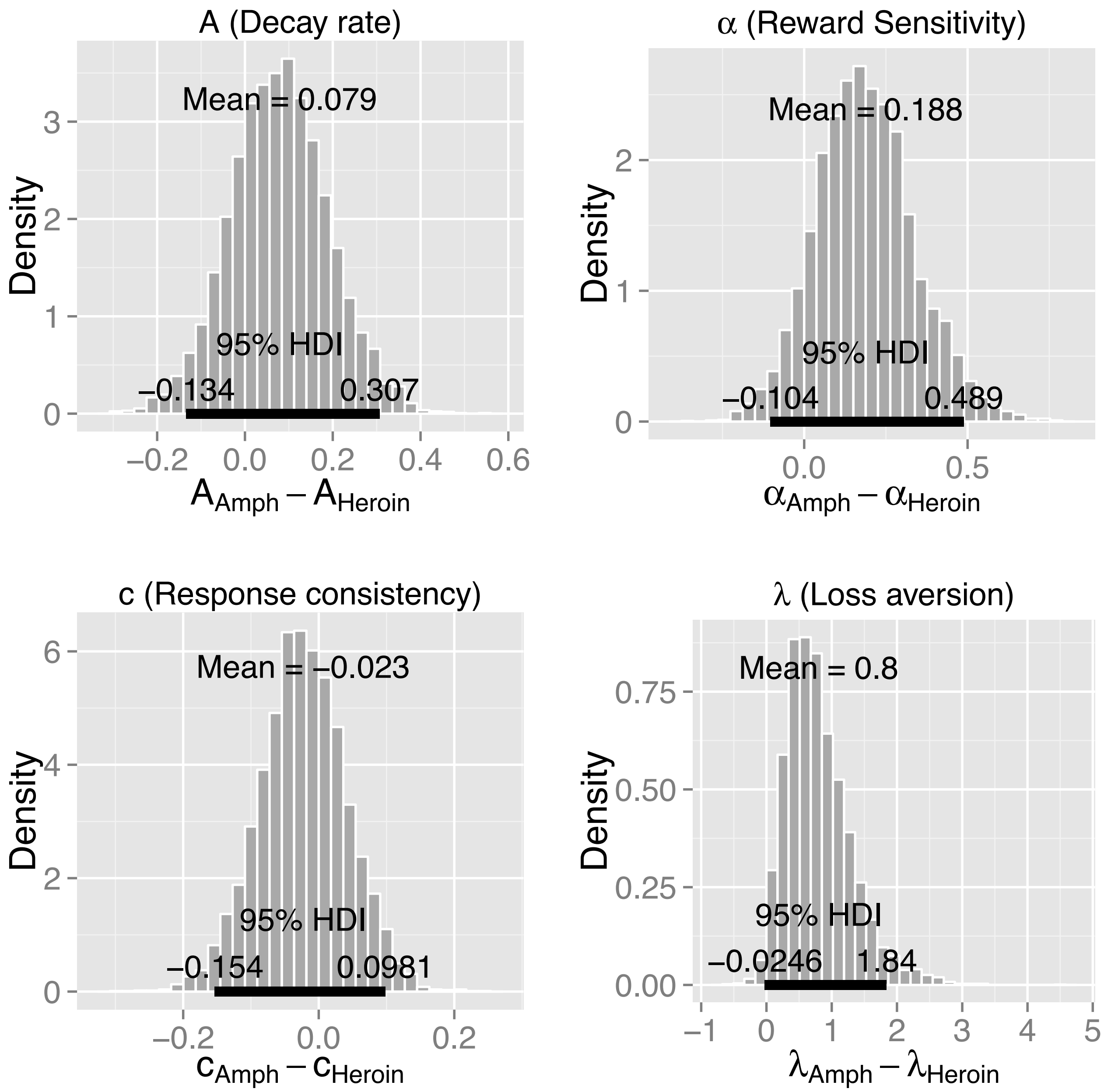

Figure S9. Posterior distributions of differences of group mean parameters between the heroin and the healthy control (HC) groups, with the PVL-Delta model. HDI = highest density interval.

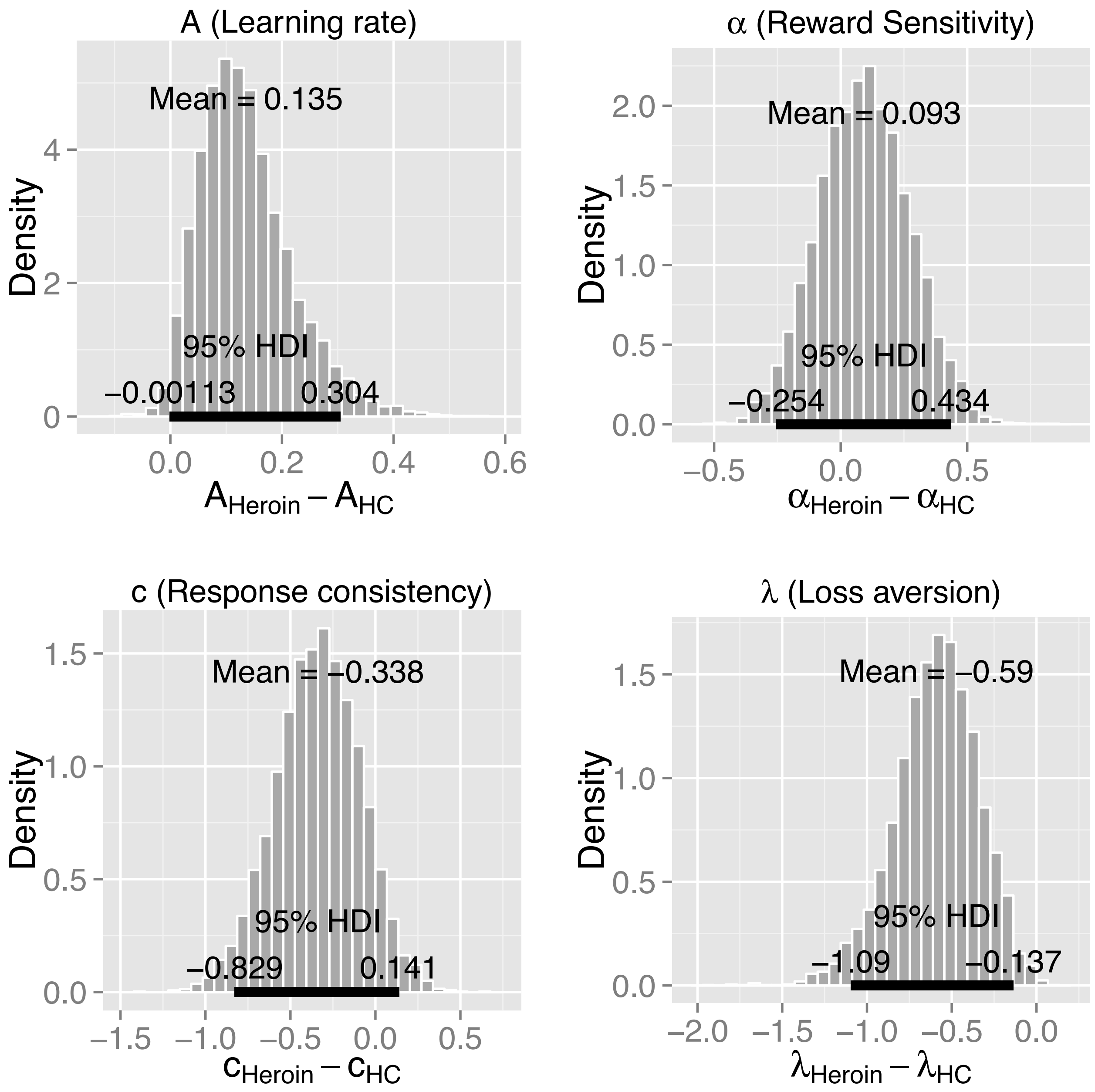

Figure S10. Posterior distributions of differences of group mean parameters between the amphetamine and the healthy control (HC) groups, with the PVL-Delta model. HDI = highest density interval.

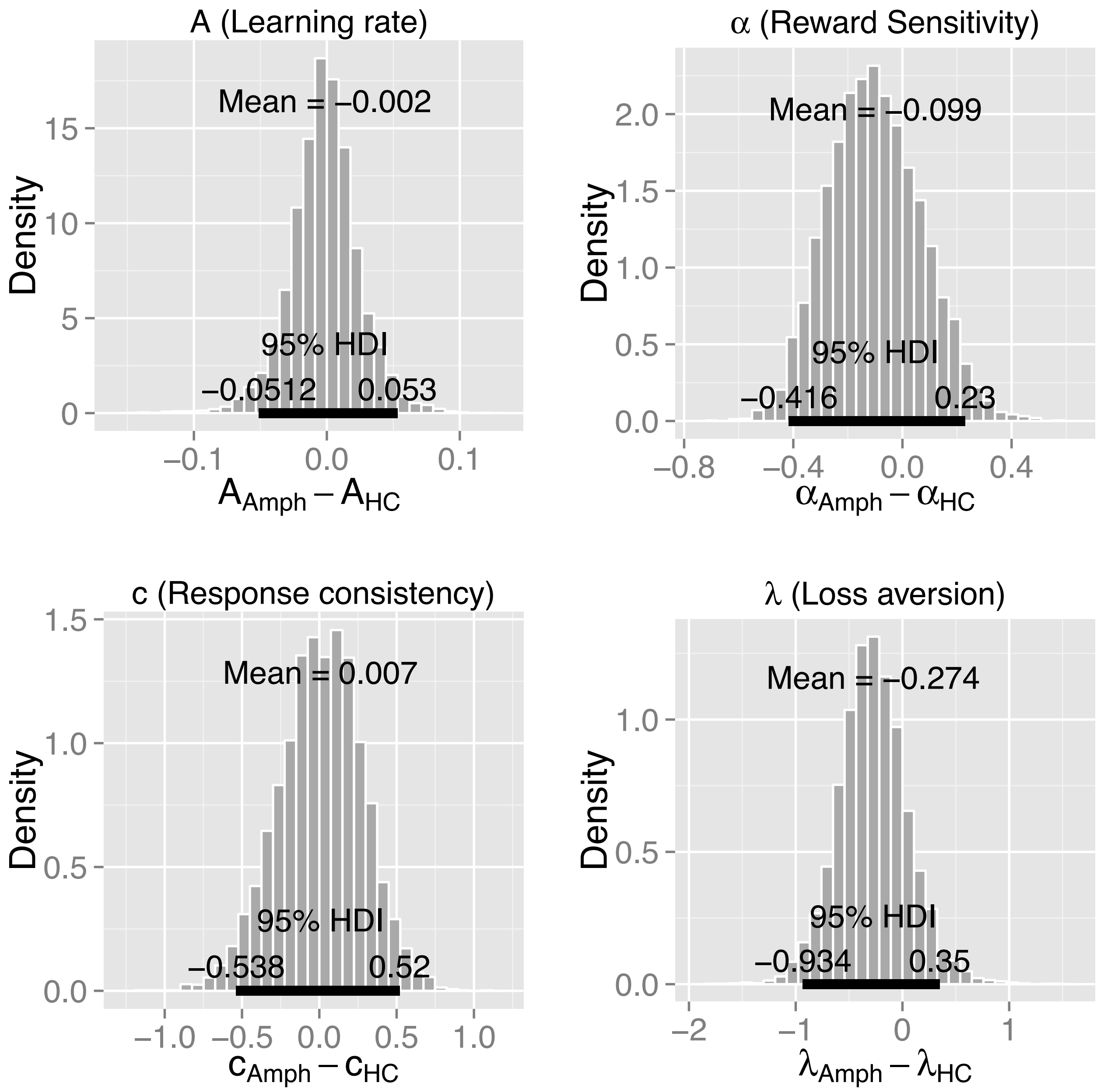

Figure S11. Posterior distributions of differences of group mean parameters between the amphetamine and the heroin groups, with the PVL-Delta model. HDI = highest density interval.

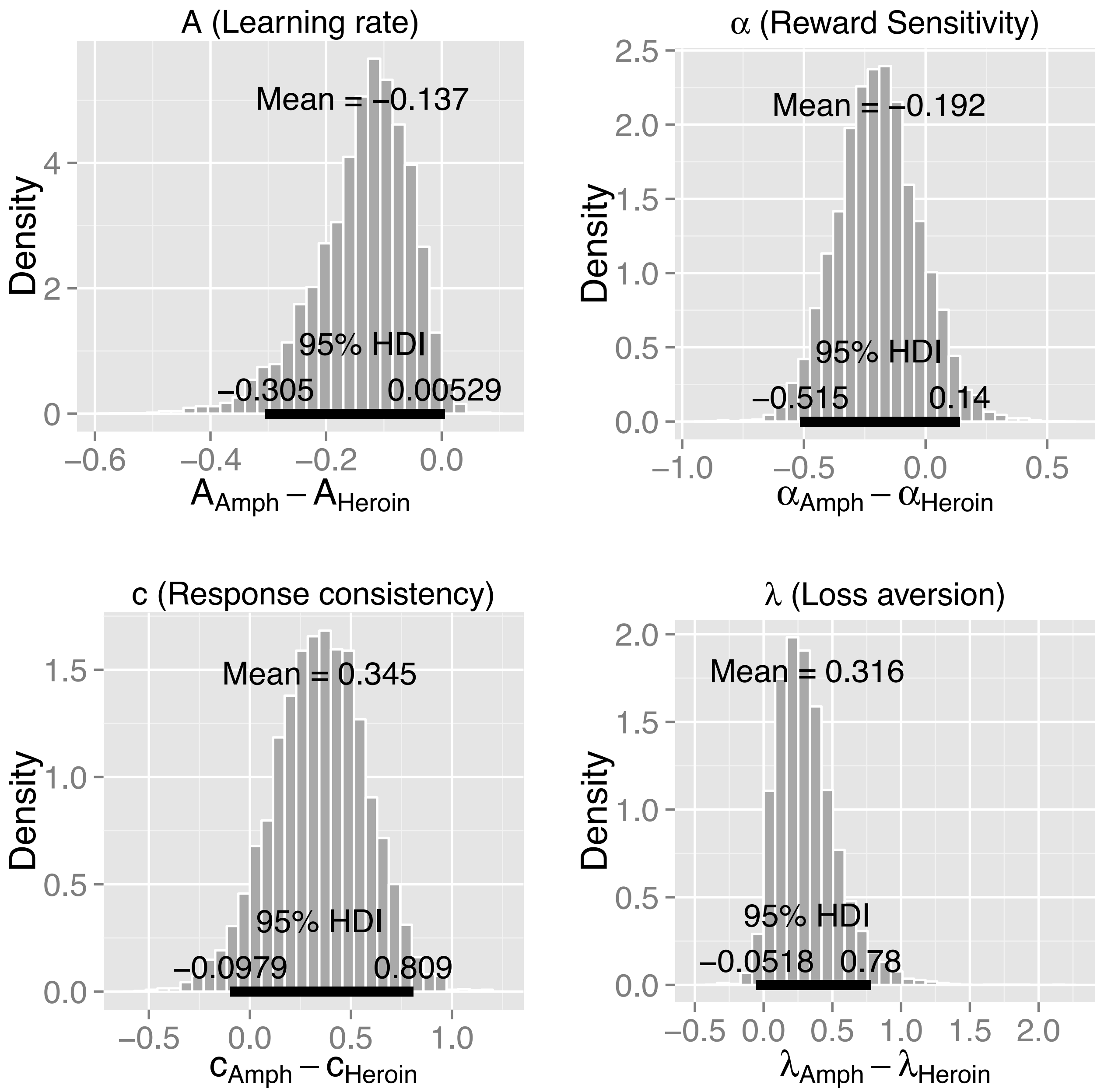

Figure S12. Posteriors for the multiple regression analysis with heroin users. Their loss aversion (λ) parameter estimated with the VPP model was the predicted variable.

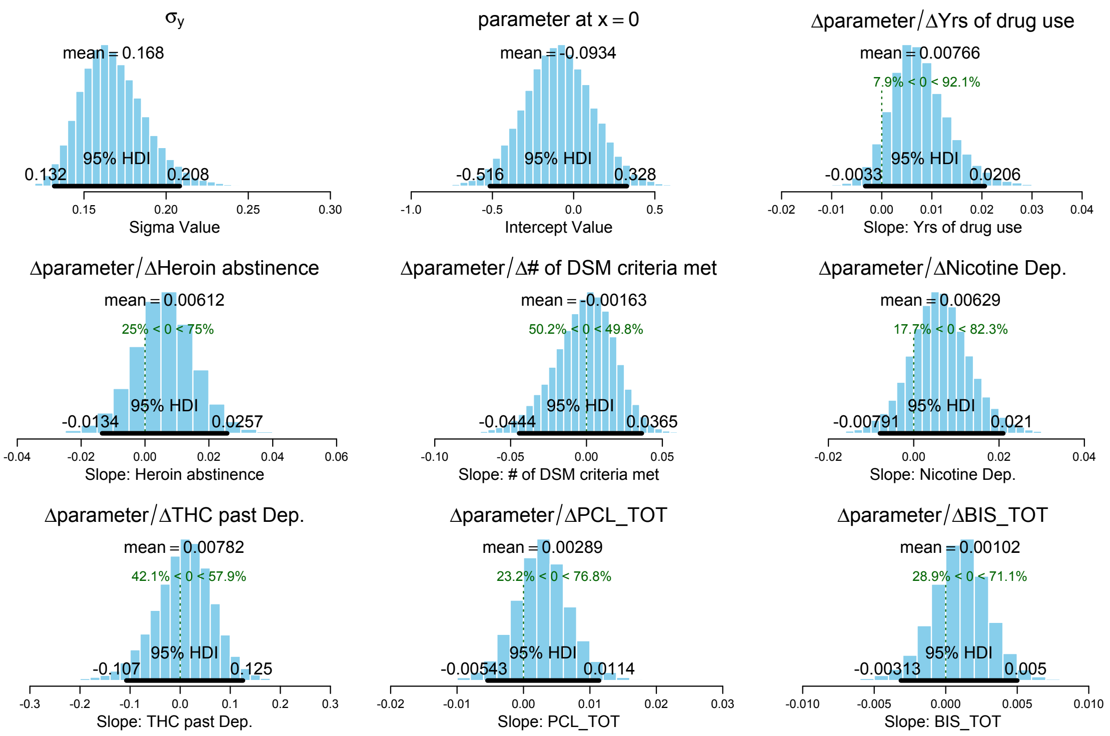

Figure S13. Posteriors for the multiple regression analysis with heroin users. Their loss aversion (λ) parameter estimated with the PVL-DecayRI model was the predicted variable.

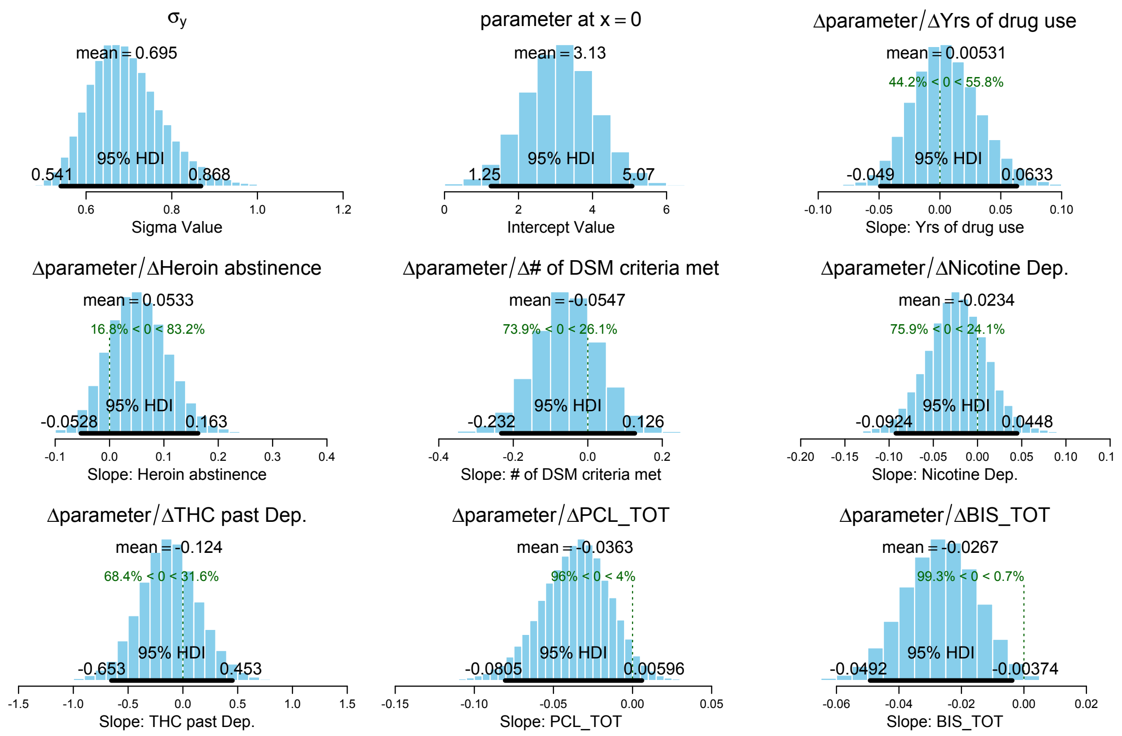

Figure S14. Posteriors for the multiple regression analysis with amphetamine users. Their reward sensitivity (α) parameter estimated with the PVL-DecayRI model was the predicted variable.

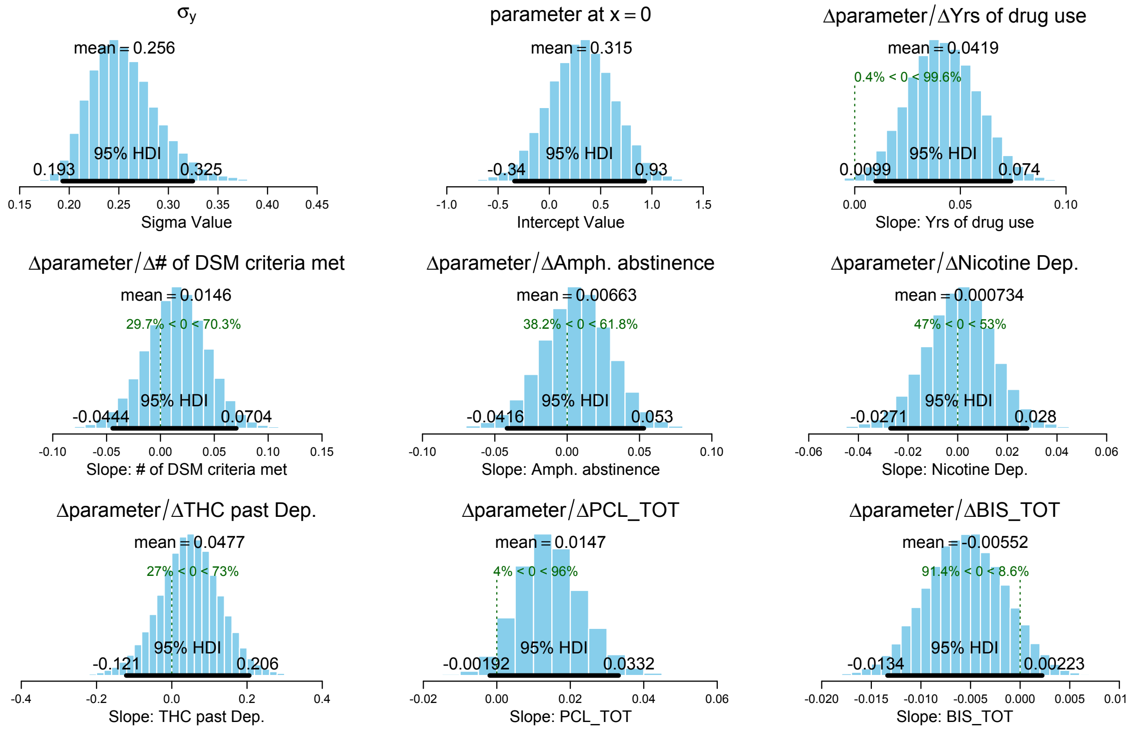

Appendix

Here, we report the results of parameter recovery results to examine how well each model can recover true parameters for synthetic data. For the purpose, we generate synthetic data that were based on the individual posterior means of the HC group by fitting each of the three models we compared (VPP, PVL-DecayRI, and PVL-Delta). Previous studies show that PVL models showed relatively good parameter recovery performance (Ahn et al., 2011 for the PVL-DecayRI and Steingroever et al., 2013 for the PVL-Delta), but the VPP model has not been tested yet with the parameter recovery method.

Figures A1, A2, and A3 show the results of the parameter recovery for the VPP, PVL-DecayRI, and PVL-Delta models, respectively. Red circles indicate true parameters of rank ordered participants (Steingroever et al., 2013) and recovered parameters were illustrated with density plots of individual posterior distributions and tick marks, which indicate mean values for each participant. Qualitatively, the PVL-Delta showed the best performance, followed by the PVL-DecayRI, and the VPP models.

Figure A1. Parameter recovery performance of the VPP model. Red circles indicate true values used to simulate synthetic data and black tick marks indicate the means of individual posterior distributions. Density plots range from 0.01% to 99.99% of posterior distributions. Participants are rank ordered based on true parameter values (red circles).

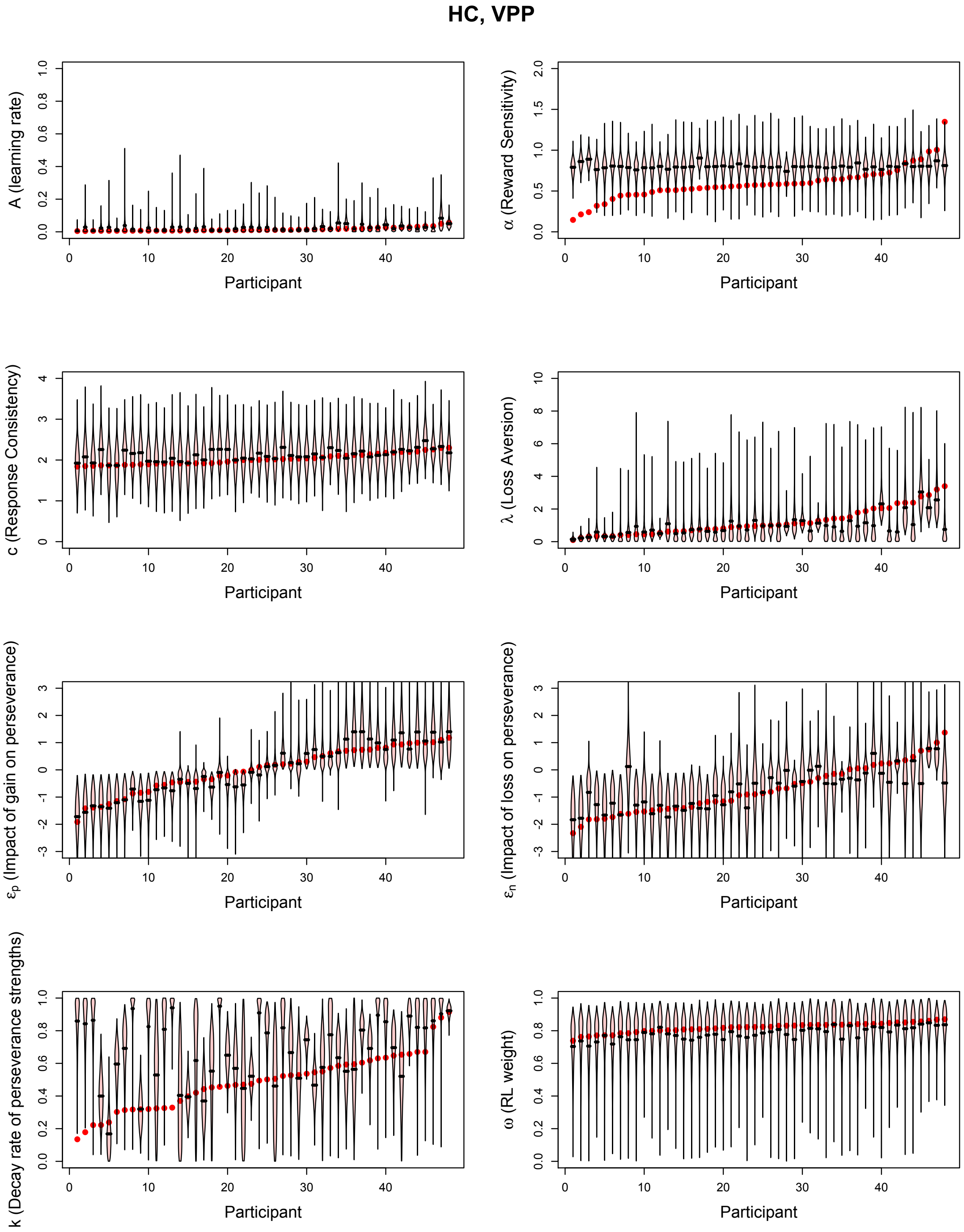

Figure A2. Parameter recovery performance of the PVL-DecayRI model. Red circles indicate true values used to simulate synthetic data and black tick marks indicate the means of individual posterior distributions. Density plots range from 0.01% to 99.99% of posterior distributions. Participants are rank ordered based on true parameter values (red circles).

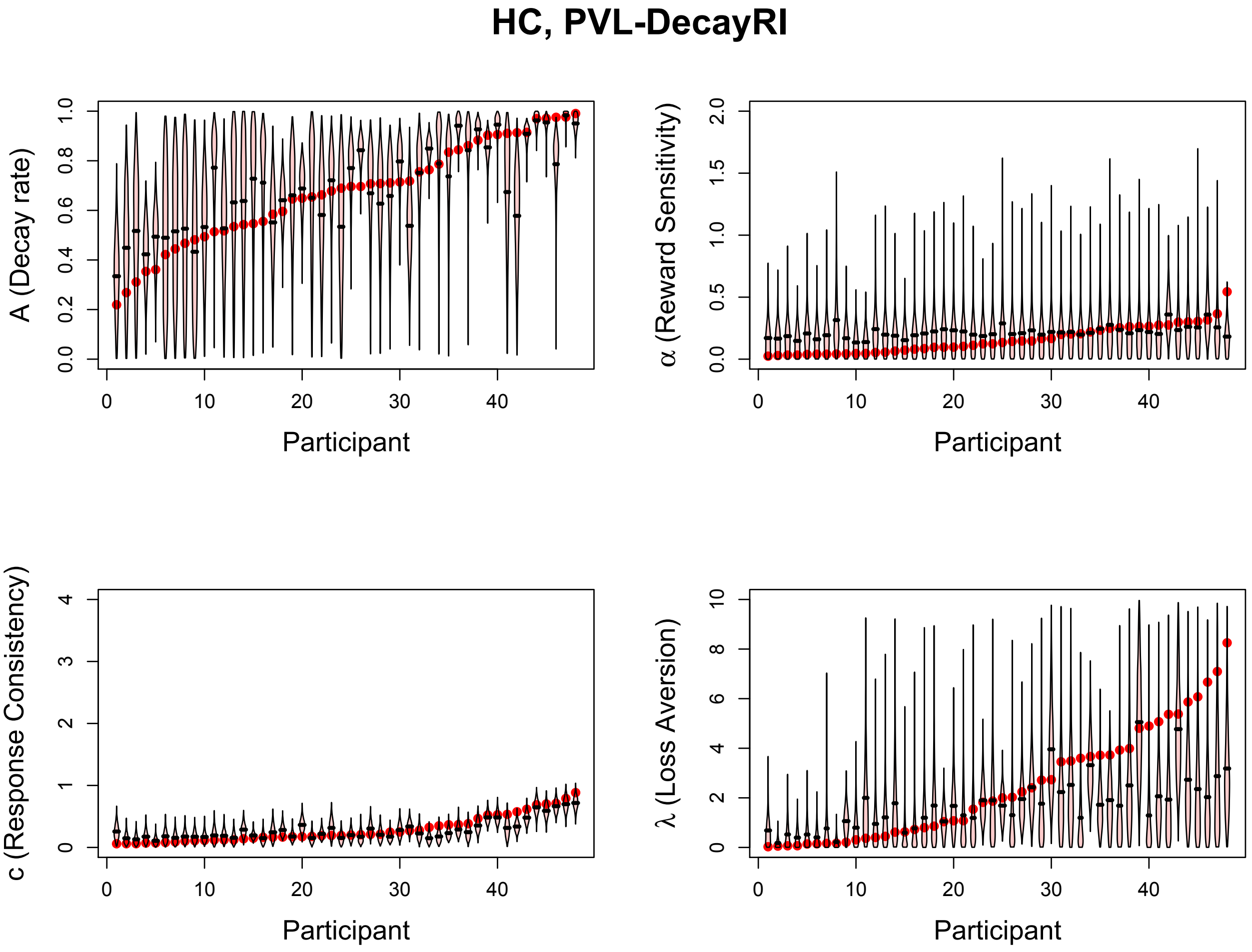

Figure A3. Parameter recovery performance of the PVL-Delta model. Red circles indicate true values used to simulate synthetic data and black tick marks indicate the means of individual posterior distributions. Density plots range from 0.01% to 99.99% of posterior distributions. Participants are rank ordered based on true parameter values (red circles).

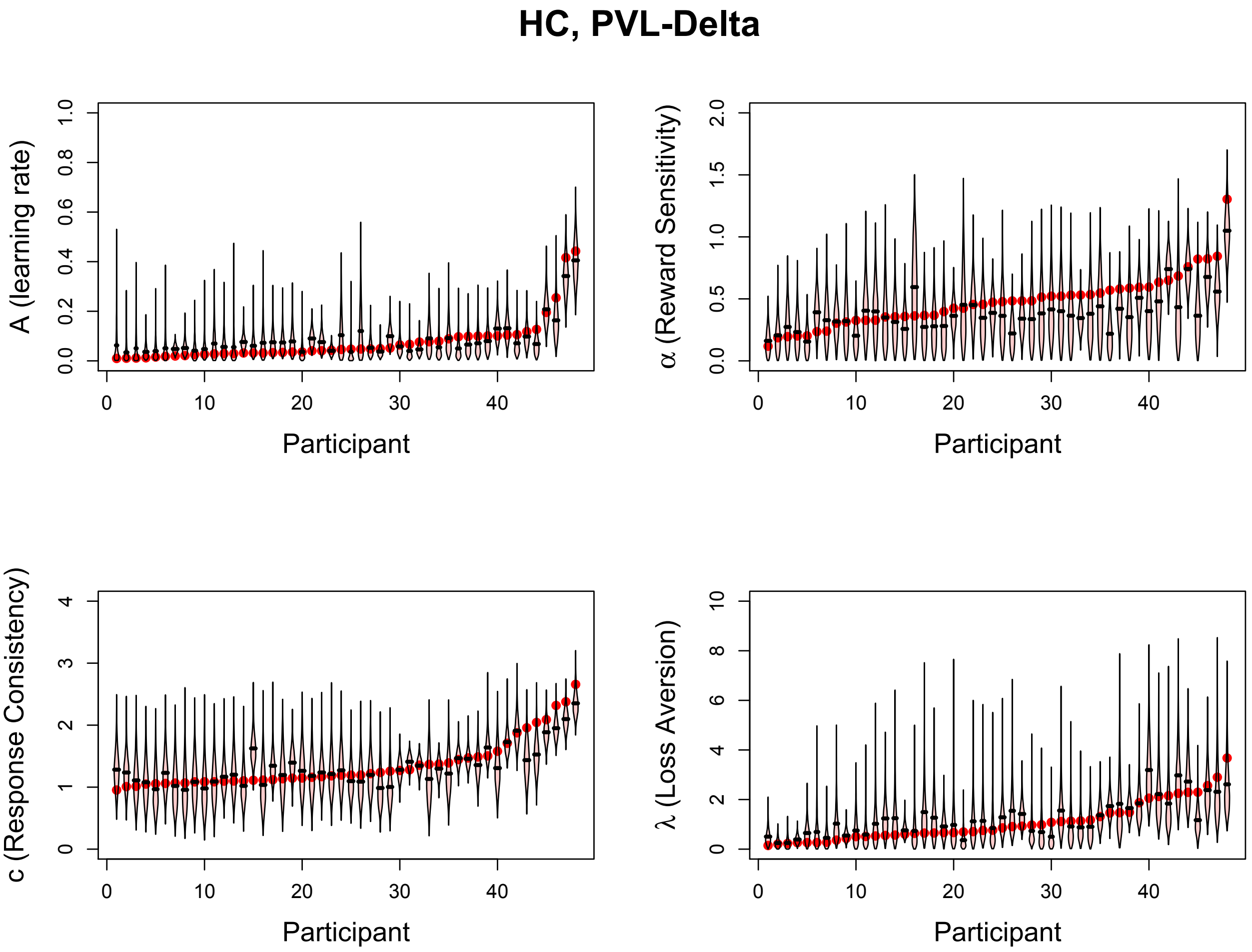

Supplementary References

Ahn, W.-Y., Krawitz, A., Kim, W., Busemeyer, J. R., and Brown, J. W. (2011). A model-based fMRI analysis with hierarchical bayesian parameter estimation. *J. Neurosci. Psychol. Econ.* 4, 95–110. doi:10.1037/a0020684

Steingroever, H., Wetzels, R., and Wagenmakers, E.-J. (2013). Validating the PVL-Delta model for the Iowa gambling task. *Front. Psychol.* 4:898. doi: 10.3389/fpsyg.2013.00898
